# Antioxidant Chimeric Molecules: Are Chemical Motifs Additive? The Case of a Selenium-Based Ligand

**DOI:** 10.3390/ijms241411797

**Published:** 2023-07-22

**Authors:** Davide Zeppilli, Anna Aldinio-Colbachini, Giovanni Ribaudo, Cristina Tubaro, Marco Dalla Tiezza, Marco Bortoli, Giuseppe Zagotto, Laura Orian

**Affiliations:** 1 Dipartimento di Scienze Chimiche, Università degli Studi di Padova, Via Marzolo 1, 35131 Padova, Italy; 2 CNRS, Aix Marseille Université, BIP, IMM, IM2B, 31 Chemin J. Aiguier, 13009 Marseille, France; 3 Dipartimento di Medicina Molecolare e Traslazionale, Università degli Studi di Brescia, Viale Europa 11, 25123 Brescia, Italy; 4 Hylleraas Centre for Quantum Molecular Sciences, Department of Chemistry, University of Oslo, 0315 Oslo, Norway; 5 Dipartimento di Scienze del Farmaco, Università degli Studi di Padova, Via Marzolo 5, 35131 Padova, Italy

**Keywords:** antioxidant, DFT calculations, gold carbene complexes, ROS scavenging, selenium, structure reactivity

## Abstract

We set up an in silico experiment and designed a chimeric compound integrating molecular features from different efficient ROS (Reactive Oxygen Species) scavengers, with the purpose of investigating potential relationships between molecular structure and antioxidant activity. Furthermore, a selenium centre was inserted due to its known capacity to reduce hydroperoxides, acting as a molecular mimic of glutathione peroxidase; finally, since this organoselenide is a precursor of a N-heterocyclic carbene ligand, its Au(I) carbene complex was designed and examined. A validated protocol based on DFT (Density Functional Theory) was employed to investigate the radical scavenging activity of available sites on the organoselenide precursor ((SMD)-M06-2X/6-311+G(d,p)//M06-2X/6-31G(d)), as well as on the organometallic complex ((SMD)-M06-2X/SDD (Au), 6-311+G(d,p)//ZORA-BLYP-D3(BJ)/TZ2P), considering HAT (Hydrogen Atom Transfer) and RAF (Radical Adduct Formation) regarding five different radicals. The results of this case study suggest that the antioxidant potential of chemical motifs should not be considered as an additive property when designing a chimeric compound, but rather that the relevance of a molecular topology is derived from a chemical motif combined with an opportune chemical space of the molecule. Thus, the direct contributions of single functional groups which are generally thought of as antioxidants per se do not guarantee the efficient radical scavenging potential of a molecular species.

## 1. Introduction

The cellular redox balance, that is, the intracellular balance of pro-oxidant and antioxidant species, is finely tuned in living beings. The deterioration of this equilibrium condition leads to oxidative stress, which is characterised by an excess of harmful free radicals [1]. It is noteworthy that these chemical species containing one or more unpaired electrons [1] are physiologically generated in biological systems, e.g., during the oxidative phosphorylation [2] or by fundamental enzymes such as cytochrome P450, mainly in the form of reactive nitrogen species (RNS) and reactive oxygen species (ROS) [3]. ROS, e.g., hydroxyl radicals (^•^OH), superoxide anion radicals (^•^O_2_^−^) and hydroperoxyl radicals (^•^OOR), are the most dangerous and contribute to severe injury [4]. Their accumulation leads to nucleic acid damage, protein modification and lipid peroxidation and has been associated with the onset and progression of several pathological conditions, e.g., neurodegenerative disorders [5], cardiovascular diseases [6] and some types of cancer [7], as well as mood disorders [8] and schizophrenia [9].

Antioxidants can contrast with oxidative stress by inhibiting radical-promoted oxidation processes [10]. They can be classified as endogenous and exogenous systems. In the former group, we find enzymes such as superoxide dismutase (SOD), glutathione peroxidase (GPx) and catalase. While SODs convert ^•^O_2_^−^ into H_2_O_2_ and H_2_O [11], GPxs [12,13] and catalases [14] transform hydroperoxides into nonradical, nonreactive and thermally stable products [15]. Among endogenous antioxidants, there are also some molecular systems, such as glutathione (GSH) [16] and melatonin [17], which exert their action by scavenging the radicals. This capacity is also found in exogenous antioxidants, which are mostly natural substances introduced with food. They include phenolic compounds, such as flavonoids [18], and dietary molecules, like tocopherols [19], carotenoids [20], omega-3 fatty acids [21], coenzyme Q10 [22], N-acetylcysteine [23], ginkgolides and bilobalide [24] and ascorbic acid [25]. In fact, in recent years, various epidemiological studies have correlated the consumption of antioxidant dietary compounds in humans with a reduced risk of developing diseases related to oxidative stress [26,27]. Interestingly, accumulating evidence also ascribes their cellular effects to their potential for modulating intracellular signalling pathways [28,29,30,31], key proteins’ activity [32] and genes [33,34], as well as the gut microbiota [34,35]. 

**Scheme 1:**
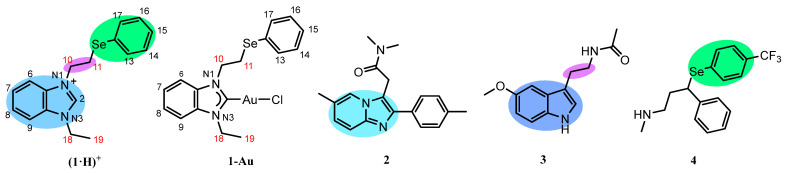
Chemical structures of the chimeric pro-ligand (1·H)^+^, the NHC-Au(I) complex (1-Au), zolpidem (2), melatonin (3) and selenofluoxetine (4). Red numbers indicate sites from which HAT may occur; black numbers indicate that RAF may occur.

All these indications have inspired physicians to complement standard therapies with antioxidant treatments for a wide range of disorders, to improve quality of life and try to slow the progression of their pathologies [36,37,38]. It is also worth noting that the central nervous system (CNS) is particularly vulnerable to oxidative stress, due to its high oxygen consumption, and antioxidant supplements can also improve patient conditions in the presence of mood disorders and dependencies [39,40]. As further evidence for this assessment, the great effectiveness of some drugs historically used for the treatment of CNS diseases has been linked to their ability to mitigate oxidative stress as a sub action of their primary pharmacological activity [41]. As proved by in silico studies, examples are the popular fluoxetine (better known by its commercial name, Prozac), a selective serotonin reuptake inhibitor (SSRI) used mainly as an antidepressant [42,43,44], and zolpidem (2, Scheme 1), a GABA_A_ receptor agonist belonging to the class of imidazopyridines, which acts as a sedative and hypnotic [45]. For the latter, the effectiveness was recently also experimentally proved by Yousefsani et al. [46]. Despite numerous in vitro and in vivo studies, the consequences of most antipsychotics and antidepressants on the cellular redox balance are still controversial and the collection of works investigating their antioxidant properties at the molecular level is scarce [41]. Therefore, research is needed to validate the proof of concept linking the complex aetiology of CNS disorders to the need for polypharmacological agents with enhanced antioxidant activity. A strategy to move forward relies on new approaches for the unambiguous characterisation of well-known drugs, together with the rational design of new drug candidates.

Computational techniques are a green, time and cost-saving tool to screen and rationalise the properties and mechanisms of molecular compounds. More than 70 commercialised drugs include some form of in silico study [47]. In particular, quantum chemistry calculations provide detailed structural, thermodynamic, and kinetic information on the elementary yet fundamental chemical reactions, but due to their computational burden and complexity, they are employed on highly specific and limited occasions in the drug discovery field. This is the case for numerous natural antioxidant substances [48], but also for bioinspired molecular compounds such as organoselenides, which are mimics of glutathione peroxidase (GPx) [49,50,51]. Density functional theory (DFT) methods are mostly used in these analyses because of their well-known accuracy at moderate computational cost. Unravelling the mechanistic features and energetics of the elementary reactions in which antioxidant activity is rooted is crucial to a rational approach to molecular design. 

In this work, we seek to investigate whether and to what extent the chemical motifs of well-known antioxidants are additive. Taking inspiration from drugs and natural compounds with known antioxidant properties, we designed a benzimidazole scaffold ‘decorated’ at position N3 with an ethyl group, and at position N1 with a group containing a selenium centre and a phenyl ring ((1·H)^+^, Scheme 1). The (1·H)^+^ was used as a precursor of the N-heterocyclic carbene (NHC) Au(I) complex 1-Au (Scheme 1). The reactivity of cation (1·H)^+^ and 1-Au was characterised according to a state-of-the-art computational protocol based on DFT calculations [44,45,52]. The radical scavenging activity of all available sites was investigated considering HAT (Hydrogen Atom Transfer) towards five different radicals (^•^OH, ^•^OCH_3_, ^•^OOH, ^•^OOCH_3,_^•^OOCH=CH_2_) and RAF (Radical Adduct Formation) with the hydroxyl radical; the capacity of the selenium centre to reduce hydrogen peroxide was also estimated. 

## 2. Results and Discussion

### 2.1. Rational Design of the Chimeric Compound

The characteristics of (1·H)^+^ were rationally chosen; the chimera consists of (i) a benzimidazole disubstituted with (ii) an ethyl group and (iii) a group containing a selenium centre. (i) The benzimidazole derivatives are clinically relevant as potential therapeutic agents for a wide range of disorders [53], including neurodegenerative diseases (e.g., Parkinson’s [54] and Alzheimer’s [55]). Since its discovery in 1944, the benzimidazole moiety has been used as a scaffold to synthesise a wide variety of compounds involved in biological processes. In the literature, many examples show antioxidant properties and a neuroprotective role [56,57]. Structure activity relationship (SAR) studies highlight the importance of the choice in tuning benzimidazole-based molecules to specific actions [58]. A similar motif formed by two condensed rings is present in zolpidem (2, Scheme 1) and in melatonin (3, Scheme 1). (ii) In (1·H)^+^, the nitrogen substituents were selected to enhance the ROS scavenging properties of the molecule, mainly through the HAT mechanisms, as shown in previous studies [59,60]. The chain with two methylene groups was chosen because it is closely analogous to 3, the compound in which it contains the most reactive site for the scavenging activity. In 2, the aromatic scaffold is connected to the nitrogen atom through two carbon atoms, one of which is involved in the amide bond. Indeed, also in 2, the methylene group at this level is the most reactive site [45]. This spacer is also common in other CNS drugs, such as fluoxetine or 4. (iii) The selenium atom was inserted in the system because of its capacity to reduce hydroperoxides [61]. In the last 40 years, many studies have proved the importance of selenium-based chemistry for human redox balance [62,63]. Selenocysteine is the key residue in the mechanism of action of several redox enzymes, including five different GPxs, thioredoxin reductases, methionine sulfoxide reductase and iodothyronine deiodinase [15]. This evidence continuously inspires the design and study of selenium-based organochalcogenides acting as GPx mimics; the most popular is ebselen (2-phenyl-1,2-benzisolenazol-3(2H)-one) [49], a selenenylamide which has been used in different clinical trials. Recently, the inclusion of one atom of selenium in fluoxetine was reported to improve its antioxidant properties [43].

Finally, (1·H)^+^ may be a precursor of a NHC ligand: the loss of the proton in C_2_ allows the formation of transition metal complexes such as the Au(I) complex 1-Au (Scheme 1). Functional NHC ligands and their gold(I) complexes are currently the subject of intense investigation in medicinal chemistry as anticancer drugs [64,65,66,67]. In this regard, the incorporation of -SeR moieties into gold(I) NHC complexes may provide compounds capable of exerting a dual action on cancer cells. Azolium salts functionalised with -SeR moieties in the pendant nitrogen group and the corresponding metal complexes are already reported in the literature, but the number of studies is still limited [68,69,70,71,72,73,74,75,76]. 

The reactivity of (1·H)^+^ was investigated in silico; its potential energy surface was explored considering two different scavenging mechanisms, hydrogen atom transfer (HAT) and radical adduct formation (RAF). Both reaction mechanisms have been characterised thermodynamically and kinetically. In addition, we have investigated the hydrogen-peroxide-reducing capability of the selenide. To point out how the presence of environments of different polarity might influence the energetics of the HAT/RAF processes, (1·H)^+^ has been studied in the gas phase, water and benzene.

**Figure 1:**
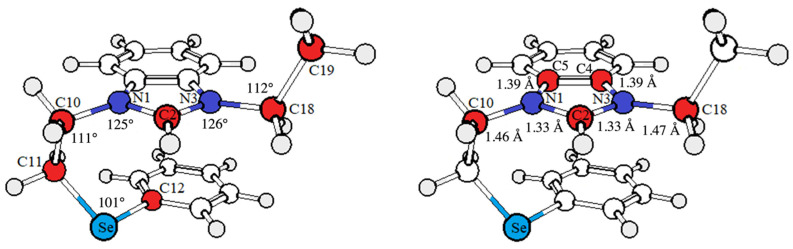
Fully optimised structure of (1·H)^+^ showing the most relevant bond angles and lengths; the atoms used in the measurements have been highlighted with colours for clarity, i.e., C (red), N (blue), Se (cyan). Level of theory: M06-2X/6-31G(d).

First, the geometry of (1·H)^+^ was fully optimised in the gas phase. Selected bond angles and lengths are shown in Figure 1. In particular, the imidazole and benzene rings are stacked in this structure. We confirmed that this arrangement corresponds to the lowest energy minimum with a scan analysis of the dihedral N1-C10-C11-Se.

### 2.2. HAT Scavenging Mechanism of (1·H)^+^

Hydrogen atom transfer (HAT) is a concerted transfer of a hydrogen atom [77], i.e., ^•^H, in a single kinetic step, from an H atom donor (e.g., (H_n_L^+^)) to an acceptor (e.g., a ROS (^•^R)), according to Equation (1):

H_n_L^+^ + ^•^R → H_n−1_L^•+^ + HR (1)

In this work, we have considered five ROSs (^•^R = ^•^OH, ^•^OCH_3_, ^•^OOH, ^•^OOCH_3_, ^•^OOCH=CH_2_). The hydroxyl radical (^•^OH) is the most electrophilic and reactive among the oxygen-centred radicals, with a half-life of ∼10^−9^ s [78]; its high reactivity is accompanied by a low selectivity. Since metal ions, particularly iron, are widely present in biological systems, ^•^OH formation is mainly attributed to the so-called iron-catalysed Haber–Weiss reaction [79,80], which makes use of Fenton chemistry (H_2_O_2_ + ^•^O_2_^–^ $\overset{{Fe}^{3+}}{\to }$ OH^–^ + ^•^OH + O_2_). In cellular environments, oxy radicals [81] are also present. The simplest is the methoxy radical (^•^OCH_3_). Peroxyl radicals (such as ^•^OOH; ^•^OOCH_3_; ^•^OOCH=CH_2_) are less reactive species, capable of diffusing to remote cellular locations; their half-lives are of the order of seconds [82]. ^•^OOH and ^•^OOCH_3_ were also chosen because they are the peroxyl analogues of ^•^OH and ^•^OCH_3_; ^•^OOCH=CH_2_ is used as a mimic of lipid peroxyl radicals. 

**Figure 2:**
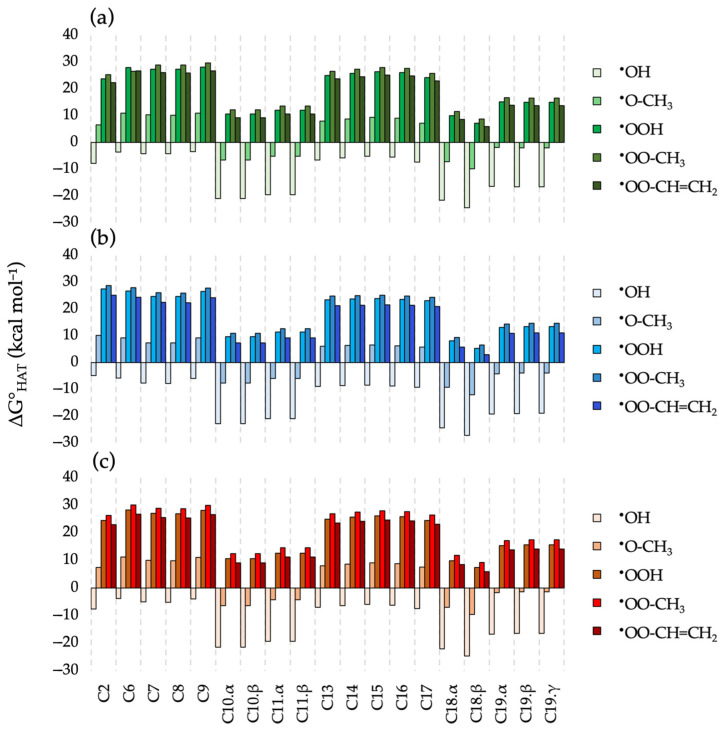
ΔG°_HAT_ (kcal mol^−1^) in gas phase (**a**), in water (**b**) and in benzene (**c**) for the scavenging of ^•^OH, ^•^OCH_3_, ^•^OOH, ^•^OOCH_3_ and ^•^OOCH=CH_2_ via HAT from all the available sites of (1·H)^+^. Level of theory: (SMD)-M06-2X/6-311+G(d,p)//M06-2X/6-31G(d).

The thermodynamics of the HAT mechanism were investigated first because they reveal the feasibility of the reactions. All available sites of (1·H)^+^ were screened (see Scheme 1 for numbering). Since the direction from which the radical might arrive and react is not predictable, multiple hydrogens belonging to the same site were considered (different H on the same C atom is indicated with α, β, γ). The Gibbs free reaction energies (ΔG°_HAT_) computed in the gas phase and in two different solvents, i.e., water and benzene, are shown in Figure 2; the reaction energies of the most reactive sites are reported in Table S1.

All reactions with the hydroxyl radical in the gas phase (Figure 2a) were exergonic, with ΔG°_HAT_ values in the range of −24.33 to −3.56 kcal mol^−1^. Only the most reactive sites (C10, C11, C18, C19) showed a certain reactivity with the methoxy radical; their ΔG°_HAT_ values were in the range –9.83 to 1.86 kcal mol^−1^. The other reactions with ^•^OCH_3_, as well as all reactions with hydrogen peroxyl, methyl peroxyl and the lipid peroxyl radicals, were strongly endergonic and thus thermodynamically unfavoured. The values were in the range of 7.26 to 28.12 kcal mol^−1^ (^•^OOH), 8.86 to 29.71 kcal mol^−1^ (^•^OOCH_3_) and 5.97 to 26.82 kcal mol^−1^ (^•^OOCH=CH_2_), respectively. C10, C11, C18 and C19, i.e., the alkyl sites inserted in (1·H)^+^ to mimic the most reactive sites in melatonin and zolpidem, were seen to have much lower ΔG°_HAT_ then the aromatic sites. Overall, C18 was the most reactive site for scavenging through the HAT mechanism for all radicals. When different hydrogens belong to the same site, they show similar reactivity. The greatest dissimilarity was found for C18.α and C18.β; the latter was systematically more favoured by ~3 kcal mol^−1^. The reason for this difference is probably due to steric effects; in both radicals, the C2-N3-C18-C19 dihedral was almost planar but, in the most stable configuration, it had a value close to 0°, positioning the C19 methyl group on the free side of C2. In contrast, in the other configuration, the dihedral was near 180° and the methyl group was closer to the benzene ring; thus, the steric hindrance was greater. In water (Figure 2b), an appreciable stabilisation of the products occurred, while in benzene (Figure 2c) the values were very close to those computed in the gas phase; thus, the trends in the gas phase were also maintained in solvent. Only in the case of C2 does the HAT mechanism generate a product which is more stable in the gas phase and in benzene than in water.

A kinetic analysis of the HAT reaction from each site was carried out in the gas phase, water and benzene (Table 1). In the gas phase, activation barriers spanned a range that reached 5.71 kcal mol^−1^ in the case of C11.β. In water and benzene, this value was seen to be higher, with the least favourable reactions being at C10.β for both solvent media with an activation energy of 4.69 and 5.32 kcal mol^−1^, respectively. HAT from site C11.α had the lowest barrier in all conditions. In the gas phase, the TS had a lower energy than the free reactants (the calculated barrier with respect to the reactant complex was 5.47 kcal mol^−1^), whereas in water and benzene very low barriers of 1.39 and 0.16 kcal mol^−1^ were computed. Interestingly, the most kinetically favoured reactions (C11.α, C19.γ) did not correspond to the most thermodynamically favoured ones (C18). Generally, the trends for the different sites were the same in all three conditions (the coordinates of the HAT radical products and the transition states are reported in Table S5 and Table S6).

**Figure 3:**
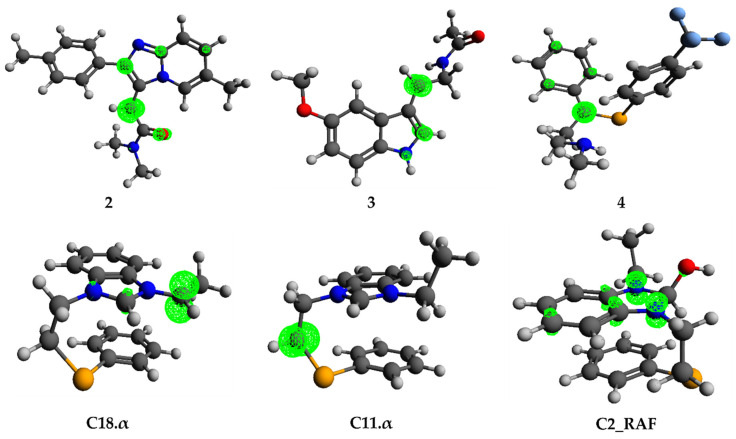
Spin densities on the radical products of 2, 3, 4 (when HAT occurs from the most reactive site) [42,45] and (1·H)^+^ when HAT occurs from C18.α and C11.α, and when RAF occurs from C2. Isodensity value of 0.015. Level of theory: M06-2X/6-311+G(d,p)//M06-2X/6-31G(d).

**Scheme 2:**
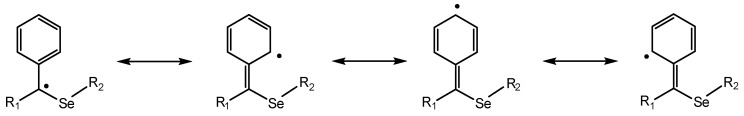
Resonance structures for the HAT radical product of a 4-like chemical structure.

These results can be compared with those obtained for 2, 3 and 4, which inspired the molecular structure of (1·H)^+^. The thermodynamic data of the HAT mechanism for these three compounds were calculated in a previous work by some of us with the same level of theory employed here [42,45]; thus, a quantitative comparison is possible. Their most reactive sites were inserted into the chemical scaffold of (1·H)^+^ and are still associated with the most favoured HAT reactions. However, the great exergonicity of HAT in 2, 3 and 4 (ΔG°_HAT_ < −30 kcal mol^−1^ for the scavenging of ^•^OH) was poorly reproduced in (1·H)^+^ and the ability to quench peroxyl radicals was completely lost. In other words, the radical products of (1·H)^+^ were not as stabilised as in the parent molecules. This outcome can be rationalised by inspecting the electronic structure of the radical products. Figure 3 shows the spin densities of the most stable radical products formed by HAT from (1·H)^+^, 2, 3 and 4. In the parent molecules, the unpaired electron was delocalised on the aromatic ring adjacent to the reaction site, particularly in zolpidem. In contrast, in the selected radicals of (1·H)^+^ (C18.α and C11.α), the spin densities were essentially localised in the carbon atom which had lost the hydrogen atom. In particular, the reactivity of the methylene group in C18 decreased in (1·H)^+^ due to a smaller delocalisation in the aromatic ring. Not even the presence of the Se nucleus seemed to improve the scavenging activity of (1·H)^+^, since there was no aromatic ring on which the spin density can be delocalised, like in 4. Scheme 2 shows how the unpaired electron is delocalised in a 4-like compound using resonance structures, according to the result of the spin density. The delocalisation involves the entire aromatic ring; therefore, in the absence of a direct phenyl group, the unpaired electron remained localised on a single carbon atom, as in (1·H)^+^. This is clear evidence that the effectiveness of an antioxidant moiety in HAT is not governed by the sole chemical nature of the atom involved in the transfer, but also requires a favourable extended structure that can efficiently delocalize the newly formed radical. Thus the ‘blind’ addition of multiple favourable HAT sites, taken from different scavengers, not only does not result in an overall better antioxidant, but could also perform worse than the distinct parent molecules.

### 2.3. RAF Scavenging Mechanism of (1·H)^+^

Radical adduct formation (RAF) is a key scavenging mechanism for molecules characterised by unsaturated bonds: electrophilic radicals are more likely to be involved in RAF reactions than non-electrophilic ones. The mechanism for a general scavenger (H_n_L^+^) is shown in Equation (2):

H_n_L^+^ + ^•^R → [H_n_L−R]^•+^ (2)

**Figure 4:**
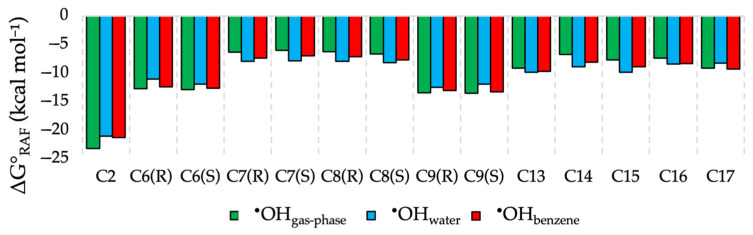
ΔG°_RAF_ (kcal mol^−1^) in gas phase (green), in water (light blue) and in benzene (red) for the scavenging of ^•^OH through RAF from all the available sites of (1·H)^+^. Level of theory: (SMD)-M06-2X/6-311+G(d,p)//M06-2X/6-31G(d).

RAF was only investigated for the hydroxyl radical. First, the thermodynamic viability of RAF reactions was determined for all the available sites (see Scheme 1). Specifically, RAF from the C6, C7, C8 and C9 sites depends on where the radical attack occurs, that is, above or under the benzimidazole plane, generating two chiral products. Gibbs free reaction energies (ΔG°_RAF_) were calculated for all stereoisomers. All the ΔG°_RAF_, calculated in the gas phase and in two different solvents, that is, water and benzene, are shown in Figure 4. The reaction energies of the most reactive sites (C2, C6, C9) are also reported in Table S2. All reactions are exergonic regardless of the environment.

Focusing on the most reactive sites, an appreciable stabilisation of the product is predicted in the gas phase (with ΔG°_RAF_ in the range −23.17 to −13.46 kcal mol^−1^) and in benzene versus water. Chiral products from the same reaction site have similar Gibbs free energies. The ΔG°_RAF_ values for the most reactive site (C2) were slightly more negative than the most favoured ones obtained for the HAT mechanism. The high reactivity via RAF from C2 can be explained by analysing the spin density of the radical product (Figure 3). In this radical, the spin density was delocalised on the two condensed rings, explaining its stability. Nevertheless, this delocalisation was not sufficient to provide the same scavenging potential predicted for 2, 3 and 4 via HAT from their most reactive sites. 

The transition states energies for RAF from C2 were calculated in the gas phase, water and benzene (the results are summarised in Table 2). Activation energies were found to be the lowest in the gas phase, as was the case for reaction energies. The energy barriers were close to those calculated for the scavenging mechanism via HAT from C19.γ and C18.β, indicating that both mechanisms may operate with the same probability at different sites of the molecule.

**Scheme 3:**
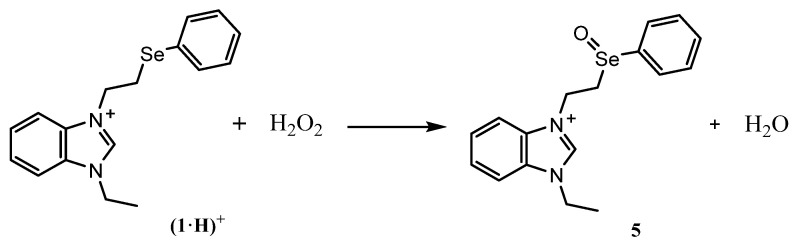
Oxidation of (1·H)^+^ by hydrogen peroxide with the formation of the selenoxide 5 and water.

The characterisation of (1·H)^+^ was completed by analysing the oxidation properties of the selenium centre. According to the literature, in the presence of hydroperoxides, selenides are mainly oxidised to selenoxides [61]; thus, a such reaction was investigated (Scheme 3).

Free reaction energies (ΔG_ox_) were calculated in the gas phase, water and benzene. In the gas phase, the barrier (ΔG^‡^_ox_) reached 48.87 kcal mol^−1^, while the stabilised P lay at −17.47 kcal mol^−1^. The reaction was undoubtedly thermodynamically favoured, but the activation energy value indicated that it is kinetically unfeasible under standard conditions. The trend in the gas phase was maintained in solution, where the reaction was more favoured both thermodynamically and kinetically. In benzene, the barrier was 47.24 kcal mol^−1^ and the energy of the free products was −22.19 kcal mol^−1^. An appreciable stabilisation of both TS (ΔG^‡^_ox_ = 38.80 kcal mol^−1^) and P (ΔG_ox_ = −35.08 kcal mol^−1^) was observed in water. These findings show that the presence of Se alone is not enough to grant an effective peroxide-reducing molecule, and a more tailored design of the chemical environment around the Se atom is necessary to arrive at a functional molecule.

### 2.4. HAT Scavenging Mechanism of 1-Au

The geometry of 1-Au was fully optimised and the selected bond angles and lengths are shown in the Supporting Information (Figure S1). A conformational analysis based on rotation about the N1-C10-C11-Se dihedral angle revealed that, like in (1·H)^+^, this structure is stabilised by dispersive inter-ring π–π interaction (Supporting Information, Figure S2).

**Figure 5:**
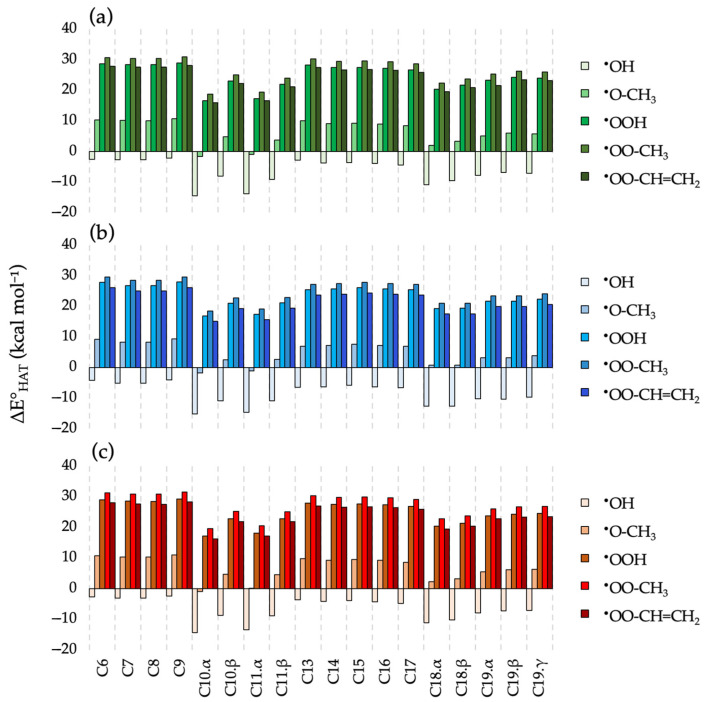
ΔE°_HAT_ (kcal mol^−1^) in gas phase (**a**), in water (**b**) and in benzene (**c**) for the scavenging of ^•^OH, ^•^OCH_3_, ^•^OOH, ^•^OOCH_3_ and ^•^OOCH=CH_2_ via HAT from all the available sites of 1-Au. Level of theory: (SMD)-M06-2X/SDD (Au), 6-311+G(d,p)//ZORA-BLYP-D3(BJ)/TZ2P.

As well as for (1·H)^+^, the capacity of scavenging through the HAT mechanism has also been investigated for 1-Au, to establish where and how the presence of gold influences the reactivity of the complex. Once again, the thermodynamic viability of the HATs was investigated first. All possible sites which might react via HAT have been screened (see Scheme 1 for numbering). Electronic reaction energies (ΔE°_HAT_) computed in the gas phase, water, and benzene are shown in Figure 5; the reaction energies of the most reactive sites are also reported in Table S3. Due to the presence of the metal centre and, therefore, the need to use a different level of theory for molecular optimisations, obtaining thermodynamic corrections would require the combination of two different functionals in order to be consistent with the level of theory used for the pro-ligand’s HAT. This approach could worsen the accuracy of the final results; hence, no Gibbs free reaction energies are reported for the mechanism of 1-Au.

The data of the gas phase (Figure 5a) show that all the reactions with the hydroxyl radical are thermodynamically favoured, as predicted for (1·H)^+^, with ΔE°_HAT_ in the range of −14.43 to –2.14 kcal mol^−1^. The most reactive sites with ^•^OH (C10.α, C11.α) showed a weak reactivity with the methoxy radical; their ΔE°_HAT_ values were in the range of −1.56 to −0.88 kcal mol^−1^. On the other hand, the remaining reactions with ^•^OCH_3_, as well as all reactions with the peroxyl radicals, were strongly endergonic and, thus, thermodynamically disfavoured. The values ranged from 16.70 to 28.99 kcal mol^−1^ (^•^OOH), 18.75 to 31.03 kcal mol^−1^ (^•^OOCH_3_) and 15.93 to 28.22 kcal mol^−1^ (^•^OOCH=CH_2_). When multiple hydrogens are on the same site, they have different tendencies to react (with the exception of the C19 site). HAT from C10.α, C11.α and C18.α sites were ∼6, ∼5 and ∼1 kcal mol^−1^ more favoured than their β correspondents, respectively. Therefore, the presence of a gold atom in the molecule actually modifies the reactivity of the organic part compared to the parent pro-ligand; in fact, all hydrogens labelled with α were further from the metal centre than their β correspondents. In particular, the reactivity of HAT of 1-Au was much lower than (1·H)^+^; the ΔE°_HAT_ for the most reactive sites of 1-Au were much less negative than the ΔE°_HAT_ of (1·H)^+^ (Supporting Information, Table S4). Moreover, the most reactive site for RAF (C2) was completely lost, due to coordination with Au(I). 

As a general rule, in water (Figure 5b), a notable stabilisation of the products was observed (with ΔE°_HAT_ in the range of –15.15 to –4.04 kcal mol^−1^ for the removal of ^•^OH), while in benzene (Figure 5c), the values were very close to those computed in the gas phase (with ΔE°_HAT_ in the range of –14.31 to –2.34 kcal mol^−1^ for the removal of ^•^OH). The trends in the gas phase were maintained in the presence of solvent, with the exception, in water, of C18, where both α and β sites had the same reactivity.

## 3. Materials and Methods

All the systems have been described at the same level of theory, except for the Au(I) complex (see below). In the absence of metal, the density functional theory (DFT) calculations were performed using Gaussian16 [84]. Full geometry optimisations were carried out using the M06-2X functional [85] with a standard 6-31G(d), a double-ζ and single polarisation basis set (level of theory: M06-2X/6-31G(d)). Three single-point energy calculations were then carried out to better estimate the electronic energies. The first single point was run in gas phase, while in the remaining two cases the SMD (Solvation Model based on Density) model [86] was used to estimate solvation effects in two different media, that is, water and benzene, mimicking a polar and an apolar environment, respectively. The basis set in these single-point calculations was extended to a 6-311+G(d,p), which is a triple-ζ quality basis set supplemented with diffuse functions and includes a double polarisation function on each atom. This level of theory is referred to as: (SMD)-M06-2X/6-311+G(d,p)//M06-2X/6-31G(d). 

The gold complex required a dedicated protocol, mainly due to the presence of the heavy metal centre; to accurately estimate its geometry, Amsterdam Density Functional (ADF) was used [87], which allows the employment of the Zeroth-order regular approximation (ZORA) to include relativistic effects in the calculations [88]. Full geometry optimisations were performed using the BLYP functional [89,90], with the inclusion of Grimme dispersion with the Becke–Johnson damping function [91,92]. The TZ2P basis set was used, that is, a large, uncontracted set of Slater-type orbitals of triple-ζ quality, augmented with two sets of polarisation functions per atom; furthermore, the small frozen core approximation was used (level of theory: ZORA-BLYP/TZ2P). Then, single-point calculations were carried out using the approach adopted for the pro-ligand described above. The only difference was the basis set associated with the gold atom, i.e., SDD [93] (Stuttgart–Dresden–Bonn optimised basis set and fully relativistic electronic core potential); the level of theory is indicated by (SMD)-M06-2X/SDD (Au), 6-311+G(d,p)//ZORA-BLYP-D3(BJ)/TZ2P.

Equilibrium and transition state geometries were optimised without symmetry constraints, using modern consolidated analytical gradient techniques. Frequency calculations were employed to calculate thermodynamic corrections at 298.15 K and 1 atm, and to verify the nature of all structures. Transition states were confirmed with the presence of a single imaginary frequency, whereas only real frequencies were computed for the minima. Spin contamination was checked for doublet ground-state species and was found to be negligible. Spin density surfaces were drawn for selected structures with Avogadro [94], with an isodensity value of 0.015.

## 4. Conclusions

This work aimed at the design of a chimeric molecule (1·H)^+^ structurally related to the well-known antioxidants 2, 3 and 4 with enhanced activity, e.g., capable of acting as a ROS scavenger via the HAT or RAF mechanisms, as well as being able to reduce hydroperoxides thanks to the presence of a selenium atom; furthermore, the presence of a Se atom could improve the overall scavenging activity, as was reported for fluoxetine and its analogous selenofluoxetine [42]. Finally, the design of the Au(I) carbene complex of (1·H)^+^ (1-Au) was employed to investigate the effect of the presence of this heavy metal centre on the scavenging activity, as well as on the capacity of selenium to reduce H_2_O_2_. 

The results presented here, obtained in silico using state-of-the-art consolidated computational methodologies, suggest that the scavenging activity of (1·H)^+^ is overall lower than that of the parent molecules. In addition, the reactivity of the selenium centre was reduced in comparison to model phenyl alkyl selenides. Moreover, the transformation of the (1·H)^+^ ligand into a N-heterocyclic carbene through the addition of an Au atom in the structure resulted in less favourable reaction energies for the HAT mechanism, both in the gas-phase and in water, when compared to (1·H)^+^, and the loss of the most reactive site for RAF because of the coordination of C2 to Au.

Although in vitro reactivity tests are needed to fully clarify these predictions, this analysis clearly demonstrates the importance of a large molecular topology for the antioxidant mechanisms, as subtle structural changes imply significant variations in reactivity, which can be nicely rationalised with modern and high-performance computational protocols. In particular, the scavenging potential does not seem to be related to localised organic moieties, i.e., functional groups or residues, but is rather tuned by the chemical environment in which the reactive site is immersed. The size of this environment remains uncertain and deserves to be explored, as does the definition of antioxidant motifs which maintain their properties when inserted in a different chemical structure.

Overall, this study demonstrates the relevance of advanced computational techniques in guiding the rational design of antioxidant molecules. However, even if it may seem counterintuitive to some extent, the concept of antioxidant motifs should overcome that of functional groups. In fact, in the case study here presented, we showed that the contributions of different and chemically diverse antioxidant ‘building blocks’ were not additive, and this aspect should be considered when the rational design of an antioxidant compound is approached, and also when using large scale methods such as those based on artificial intelligence. Thus, a wider, computer-guided exploration of chemical space may pave the way for the identification of synergistic interconnections between antioxidant motifs, and their definition and characterisation remains one of the challenges of contemporary rational drug design.

## Figures and Tables

**Table 1:** ΔG_HAT_ (kcal mol^−1^) of transition state (TS) and free products (P) in the gas phase, water and benzene for the scavenging of ^•^OH via HAT from the most reactive sites of (1·H)^+^. The values are given with respect to the free reactants (R). Level of theory: (SMD)-M06-2X/6-311+G(d,p)//M06-2X/6-31G(d).

	ΔG_HAT, gas phase_ (kcal mol^−1^)								
	C10.α	C10.β	C11.α	C11.β	C18.α	C18.β	C19.α	C19.β	C19.γ
R	0.00	0.00	0.00	0.00	0.00	0.00	0.00	0.00	0.00
TS	2.33	4.07	−0.88 *	5.43	3.55	1.40	3.48	5.71	0.83
P	−20.98	−20.97	−19.57	−19.57	−21.59	−24.3	−16.4	−16.55	−16.6
	ΔG_HAT, water_ (kcal mol^−1^)								
R	0.00	0.00	0.00	0.00	0.00	0.00	0.00	0.00	0.00
TS	2.38	5.32	1.39	3.89	2.97	3.35	2.61	2.93	2.49
P	−22.74	−22.73	−20.95	−20.95	−24.29	−27.1	−19.2	−18.94	−18.9
	ΔG_HAT, benzene_ (kcal mol^−1^)								
R	0.00	0.00	0.00	0.00	0.00	0.00	0.00	0.00	0.00
TS	2.32	4.69	0.16	4.59	3.53	2.34	3.31	4.40	1.93
P	−21.39	−21.38	−19.32	−19.32	−22.06	−24.62	−16.64	−16.39	−16.38

* The barrier calculated with reference to the reactant complex is 5.47 kcal mol^−1^ [83].

**Table 2:** ΔG_RAF_ (kcal mol^−1^) of transition state (TS) and free products (P) in gas phase, water and benzene for the scavenging of ^•^OH via RAF from the most reactive site of (1·H)^+^ (C2). The values are given with respect to the free reactants (R). Level of theory: (SMD)-M06-2X/6-311+G(d,p)//M06-2X/6-31G(d).

	ΔG_RAF_ (kcal mol^−1^)		
	Gas Phase	Water	Benzene
R	0.00	0.00	0.00
TS	1.17	1.63	2.21
P	−23.17	−19.13	−19.35

## Data Availability

The data used are contained within the article or in the Supplementary Materials.

## Supplementary Materials

The supporting information can be downloaded at: https://www.mdpi.com/article/10.3390/ijms241411797/s1.
