# Supplementary material for: Antioxidant Chimeric Molecules: Are Chemical Motifs Additive? The Case of a Selenium-Based Ligand
Source: Int J Mol Sci. 2023 Jul 22;24(14):11797. doi: 10.3390/ijms241411797 (PMC10380222; doi:10.3390/ijms241411797)
Supplement: Supplementary file 1 [file ijms-24-11797-s001.zip › ijms-2511785-supplementary.pdf]

# - SUPPORTING INFORMATION -

Antioxidant Chimeric Molecules: Are Chemical Motifs Additive? The Case of a Selenium-Based Ligand

Davide Zeppilli [1,†], Anna Aldinio-Colbachini [2], Giovanni Ribaudo [3,†], Cristina Tubaro [1], Marco Dalla Tiezza [1], Marco Bortoli [4], Giuseppe Zagotto [5] and Laura Orian [1,*]

1 Dipartimento di Scienze Chimiche, Università degli Studi di Padova, Via Marzolo 1, 35131 Padova, Italy
2 CNRS, Aix Marseille Université, BIP, IMM, IM2B 31 Chemin J. Aiguier, 13009 Marseille, France
3 Dipartimento di Medicina Molecolare e Traslazionale, Università degli Studi di Brescia, Viale Europa 11, 25123 Brescia, Italy
4 Hylleraas Centre for Quantum Molecular Sciences, Department of Chemistry, University of Oslo, Oslo 0315, Norway
5 Dipartimento di Scienze del Farmaco, Università degli Studi di Padova, Via Marzolo 5, 35131 Padova, Italy
* Correspondence: laura.orian@unipd.it; Tel.: +39 049 8275140
† These authors contributed equally to this work.

LIST of TABLES and FIGURES:

Table S1. Gibbs free reaction energies $\Delta G°_{HAT}$ (kcal mol^{-1}) computed in gas phase, water and benzene for the scavenging of $^{\bullet}OH$, $^{\bullet}OOH$, $^{\bullet}OCH_3$, $^{\bullet}OOCH_3$ and $^{\bullet}OOCH=CH_2$ via HAT from the most reactive sites of **(1·H)**$^+$. Level of theory: (SMD)-M06-2X/6-311+G(d,p)//M06-2X/6-31G(d)……….2

Table S2. Gibbs free reaction energies $\Delta G°_{RAF}$ (kcal mol^{-1}) computed in gas phase, water and benzene for the scavenging of $^{\bullet}OH$, via RAF from the most reactive sites of **(1·H)**$^+$. Level of theory: (SMD)-M06-2X/6-311+G(d,p)//M06-2X/6-31G(d)……….3

Figure S1. Fully optimized structure of **1-Au** labelled with the most relevant bond angles and lengths. Level of theory: ZORA-BLYP-D3(BJ)/TZ2P……….3

Figure S2. Linear scan of the potential energy surface along the N1-C11-C10-Se dihedral angle of **1-Au**. Level of theory: ZORA-BLYP-D3(BJ)/TZ2P……….3

Table S3. Electronic reaction energies $\Delta E°_{HAT}$ (kcal mol^{-1}) computed in gas phase, water and benzene for the scavenging of $^{\bullet}OH$, $^{\bullet}OOH$, $^{\bullet}OCH_3$, $^{\bullet}OOCH_3$ and $^{\bullet}OOCH=CH_2$ via HAT from the most reactive sites of **1-Au**. Level of theory: (SMD)-M06-2X/SDD (Au), 6-311+G(d,p)//ZORA-BLYP-D3(BJ)/TZ2P……….4

Table S4. Electronic reaction energies $\Delta E°_{HAT}$ (kcal mol^{-1}) computed in gas phase, water and benzene for the scavenging of $^{\bullet}OH$, $^{\bullet}OOH$, $^{\bullet}OCH_3$, $^{\bullet}OOCH_3$ and $^{\bullet}OOCH=CH_2$ via HAT from the most reactive sites of **(1·H)**$^+$. Level of theory: (SMD)-M06-2X/6-311+G(d,p)//M06-2X/6-31G(d)……….5

Table S5. Coordinates of the structures of **(1·H)**$^+$ and its HAT radicals at each available site. Level of theory: M06-2X/6-31G(d)……….6

Table S6. Coordinates and imaginary frequencies (Nimag, cm^{-1}) of the transition states of HAT and RAF (from C2 site). Level of theory: M06-2X/6-31G(d)……….14

Table S7. Coordinates of the structures of the RAF radicals at each available site of **(1·H)**$^+$. Level of theory: M06-2X/6-31G(d)……….18

Table S8. Coordinates of the structures for the Se oxidation of **(1·H)**$^+$. Level of theory: M06-2X/6-31G(d)……….24

Table S9. Coordinates of the structures of the five ROSs and their protonated forms. Level of theory: M06-2X/6-31G(d)……….25

Table S1. Gibbs free reaction energies $\Delta G°_{HAT}$ (kcal mol^{-1}) computed in gas phase, water and benzene for the scavenging of $^{\bullet}$OH, $^{\bullet}$OOH, $^{\bullet}OCH_3$, $^{\bullet}OOCH_3$ and $^{\bullet}OOCH{=}CH_2$ via HAT from the most reactive sites of **(1·H)**$^{+}$. Level of theory: (SMD)-M06-2X/6-311+G(d,p)//M06-2X/6-31G(d).

	$\Delta G_{HAT,\ gas\ phase}$ (kcal mol^{-1})				
	$^{\bullet}$OH	$^{\bullet}OCH_3$	$^{\bullet}$OOH	$^{\bullet}OOCH_2$	$^{\bullet}OOCH{=}CH_2$
**C10.α**	-20.98	-6.48	10.62	12.21	9.32
**C10.β**	-20.97	-6.47	10.62	12.21	9.32
**C11.α**	-19.57	-5.07	12.02	13.61	10.73
**C11.β**	-19.57	-5.07	12.02	13.62	10.73
**C18.α**	-21.59	-7.08	10.01	11.60	8.71
**C18.β**	-24.33	-9.83	7.26	8.86	5.97
**C19.α**	-16.36	-1.86	15.23	16.82	13.93
**C19.β**	-16.55	-2.05	15.04	16.63	13.74
**C19.γ**	-16.55	-2.04	15.05	16.64	13.75
	$\Delta G_{HAT,\ water}$ (kcal mol^{-1})				
	$^{\bullet}$OH	$^{\bullet}OCH_3$	$^{\bullet}$OOH	$^{\bullet}OOCH_2$	$^{\bullet}OOCH{=}CH_2$
**C10.α**	-22.74	-7.65	9.72	10.99	7.44
**C10.β**	-22.73	-7.65	9.73	11.00	7.44
**C11.α**	-20.95	-5.87	11.51	12.78	9.23
**C11.β**	-20.95	-5.86	11.51	12.78	9.23
**C18.α**	-24.29	-9.20	8.17	9.44	5.89
**C18.β**	-27.12	-12.04	5.34	6.61	3.06
**C19.α**	-19.22	-4.14	13.24	14.51	10.96
**C19.β**	-18.94	-3.86	13.52	14.79	11.24
**C19.γ**	-18.93	-3.85	13.53	14.80	11.25
	$\Delta G_{HAT,\ benzene}$ (kcal mol^{-1})				
	$^{\bullet}$OH	$^{\bullet}OCH_3$	$^{\bullet}$OOH	$^{\bullet}OOCH_2$	$^{\bullet}OOCH{=}CH_2$
**C10.α**	-21.39	-6.32	10.66	12.52	9.18
**C10.β**	-21.38	-6.31	10.66	12.53	9.19
**C11.α**	-19.32	-4.25	12.72	14.59	11.25
**C11.β**	-19.32	-4.25	12.73	14.59	11.26
**C18.α**	-22.06	-6.99	9.99	11.86	8.52
**C18.β**	-24.62	-9.56	7.42	9.29	5.95
**C19.α**	-16.64	-1.57	15.4	17.27	13.93
**C19.β**	-16.39	-1.32	15.65	17.52	14.18
**C19.γ**	-16.38	-1.32	15.66	17.53	14.19

Table S2. Gibbs free reaction energies $\Delta G°_{RAF}$ (kcal mol^{-1}) computed in gas phase, water and benzene for the scavenging of $^{•}OH$, via RAF from the most reactive sites of **(1·H)**$^{+}$. Level of theory: (SMD)-M06-2X/6-311+G(d,p)//M06-2X/6-31G(d).

	$\Delta G°_{RAF,gas\ phase}$ (kcal mol^{-1})	$\Delta G°_{RAF,water}$ (kcal mol^{-1})	$\Delta G°_{RAF,benzene}$ (kcal mol^{-1})
**C2**	-23.17	-21.03	-21.25
**C6(R)**	-12.73	-11.04	-12.36
**C6(S)**	-12.86	-11.92	-12.62
**C9(R)**	-13.39	-12.45	-13.03
**C9(S)**	-13.46	-11.86	-13.27

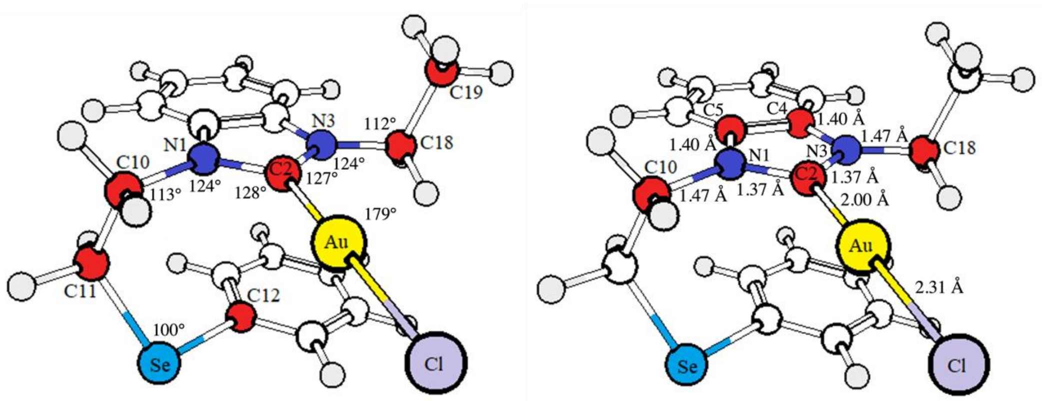

Figure S1. Fully optimized structure of **1-Au** labelled with the most relevant bond angles and lengths. Level of theory: ZORA-BLYP-D3(BJ)/TZ2P.

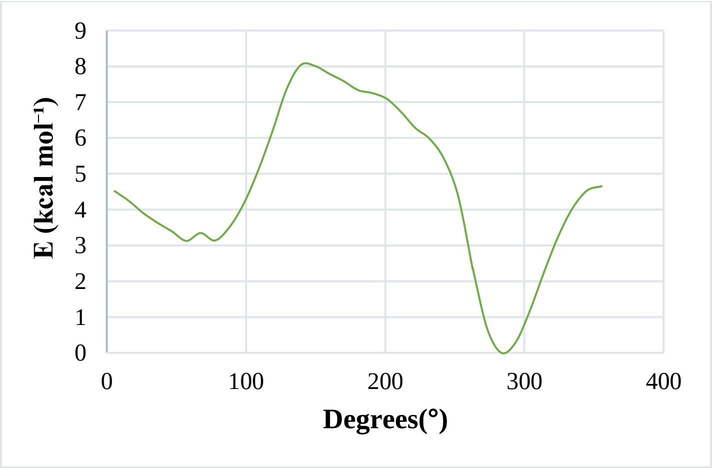

Figure S2. Linear scan of the potential energy surface along the N1-C11-C10-Se dihedral angle of **1-Au**. Level of theory: ZORA-BLYP-D3(BJ)/TZ2P.

Table S3. Electronic reaction energies $\Delta E°_{HAT}$ (kcal mol^{-1}) computed in gas phase, water and benzene for the scavenging of $^{•}OH$, $^{•}OOH$, $^{•}OCH_3$, $^{•}OOCH_3$ and $^{•}OOCH=CH_2$ via HAT from the most reactive sites of **1-Au**. Level of theory: (SMD)-M06-2X/SDD (Au), 6-311+G(d,p)//ZORA-BLYP-D3(BJ)/TZ2P.

	$\Delta E_{HAT,\ gas\ phase}$ (kcal mol^{-1})				
	$^{•}OH$	$^{•}OCH_3$	$^{•}OOH$	$^{•}OOCH_2$	$^{•}OOCH=CH_2$
**C10.α**	-14.43	-1.56	16.7	18.75	15.93
**C10.β**	-8.04	4.82	23.09	25.13	22.32
**C11.α**	-13.75	-0.88	17.38	19.43	16.62
**C11.β**	-9.16	3.71	21.97	24.02	21.21
**C18.α**	-10.78	2.09	20.35	22.4	19.58
**C18.β**	-9.44	3.43	21.69	23.74	20.92
	$\Delta E_{HAT,\ water}$ (kcal mol^{-1})				
	$^{•}OH$	$^{•}OCH_3$	$^{•}OOH$	$^{•}OOCH_2$	$^{•}OOCH=CH_2$
**C10.α**	-15.15	-1.7	16.85	18.57	15.09
**C10.β**	-10.93	2.52	21.07	22.79	19.31
**C11.α**	-14.58	-1.13	17.42	19.14	15.66
**C11.β**	-10.83	2.62	21.17	22.89	19.41
**C18.α**	-12.64	0.81	19.36	21.08	17.6
**C18.β**	-12.61	0.84	19.38	21.11	17.63
	$\Delta E_{HAT,\ benzene}$ (kcal mol^{-1})				
	$^{•}OH$	$^{•}OCH_3$	$^{•}OOH$	$^{•}OOCH_2$	$^{•}OOCH=CH_2$
**C10.α**	-14.31	-0.88	17.27	19.59	16.33
**C10.β**	-8.68	4.76	22.9	25.22	21.96
**C11.α**	-13.38	0.06	18.2	20.52	17.26
**C11.β**	-8.79	4.64	22.78	25.11	21.84
**C18.α**	-11.11	2.33	20.47	22.79	19.53
**C18.β**	-10.18	3.25	21.4	23.72	20.45

Table S4. Electronic reaction energies $\Delta E°_{HAT}$ (kcal mol^{-1}) computed in gas phase, water and benzene for the scavenging of $^{\bullet}$OH, $^{\bullet}$OOH, $^{\bullet}OCH_3$, $^{\bullet}OOCH_3$ and $^{\bullet}OOCH{=}CH_2$ via HAT from the most reactive sites of **(1·H)**$^+$. Level of theory: (SMD)-M06-2X/6-311+G(d,p)//M06-2X/6-31G(d).

	$\Delta E_{HAT,\ gas\ phase}$ (kcal mol^{-1})				
	$^{\bullet}$OH	$^{\bullet}OCH_3$	$^{\bullet}$OOH	$^{\bullet}OOCH_2$	$^{\bullet}OOCH{=}CH_2$
**C10.α**	-18.88	-6.01	12.25	14.30	11.49
**C10.β**	-18.88	-6.01	12.25	14.30	11.49
**C11.α**	-18.03	-5.16	13.10	15.14	12.33
**C11.β**	-18.03	-5.16	13.10	15.15	12.33
**C18.α**	-19.70	-6.83	11.43	13.48	10.67
**C18.β**	-22.10	-9.23	9.04	11.08	8.27
**C19.α**	-13.98	-1.11	17.15	19.20	16.39
**C19.β**	-13.98	-1.11	17.15	19.20	16.39
**C19.γ**	-13.98	-1.11	17.15	19.20	16.39
	$\Delta E_{HAT,\ water}$ (kcal mol^{-1})				
	$^{\bullet}$OH	$^{\bullet}OCH_3$	$^{\bullet}$OOH	$^{\bullet}OOCH_2$	$^{\bullet}OOCH{=}CH_2$
**C10.α**	-20.65	-7.19	11.36	13.09	9.61
**C10.β**	-20.65	-7.19	11.36	13.08	9.61
**C11.α**	-19.43	-5.96	12.59	14.31	10.83
**C11.β**	-19.43	-5.96	12.59	14.31	10.83
**C18.α**	-22.42	-8.95	9.60	11.32	7.84
**C18.β**	-24.90	-11.43	7.11	8.84	5.36
**C19.α**	-16.85	-3.38	15.16	16.89	13.41
**C19.β**	-16.38	-2.91	15.63	17.36	13.88
**C19.γ**	-16.38	-2.91	15.64	17.36	13.88
	$\Delta E_{HAT,\ benzene}$ (kcal mol^{-1})				
	$^{\bullet}$OH	$^{\bullet}OCH_3$	$^{\bullet}$OOH	$^{\bullet}OOCH_2$	$^{\bullet}OOCH{=}CH_2$
**C10.α**	-19.29	-5.85	12.29	14.61	11.35
**C10.β**	-19.29	-5.85	12.29	14.61	11.35
**C11.α**	-17.78	-4.35	13.80	16.12	12.86
**C11.β**	-17.78	-4.34	13.80	16.12	12.86
**C18.α**	-20.17	-6.73	11.41	13.73	10.47
**C18.β**	-22.39	-8.95	9.19	11.51	8.25
**C19.α**	-14.26	-0.82	17.32	19.64	16.38
**C19.β**	-13.82	-0.38	17.76	20.09	16.82
**C19.γ**	-13.82	-0.38	17.76	20.09	16.82

Table S5. Coordinates of the structures of **(1·H)**$^{+}$ and its HAT radicals at each available site. Level of theory: M06-2X/6-31G(d).

(1·H)$^{+}$

C	0.173352	-2.845573	-0.438504
C	0.870216	-2.972960	0.762220
C	-0.528767	-1.267538	1.744644
C	-1.220715	-1.135194	0.539544
C	-0.883886	-1.946980	-0.544569
H	-1.452237	-1.895794	-1.468690
Se	-2.682657	0.098012	0.482353
C	-2.418097	0.882558	-1.283836
C	-1.454446	2.070143	-1.264206
N	-0.168785	1.685698	-0.687721
C	0.777774	0.863177	-1.292139
C	1.701185	0.524894	-0.297957
N	1.273913	1.151302	0.869533
C	0.152088	1.808087	0.600902
C	1.962835	1.098779	2.164538
C	3.201873	1.983811	2.178934
H	2.934297	3.031378	2.021371
H	3.699597	1.896866	3.147215
H	3.910827	1.686496	1.401931
H	2.203166	0.047034	2.342965
H	1.238396	1.405218	2.923135
C	2.783712	-0.316784	-0.554644
C	2.891212	-0.802000	-1.847063
C	1.960976	-0.459857	-2.849284
C	0.888293	0.378749	-2.595785
H	-1.268189	2.453007	-2.272014
H	-1.854252	2.886429	-0.659172
H	-2.079978	0.108942	-1.975789
H	-3.402730	1.220934	-1.612151
H	0.435091	-3.466489	-1.289373
H	-0.810041	-0.652443	2.596556
C	0.506011	-2.194813	1.858176
H	1.018984	-2.314160	2.808302
H	1.678431	-3.691980	0.848466
H	3.496866	-0.585502	0.217185
H	2.092374	-0.862528	-3.847589
H	3.714264	-1.462137	-2.097403
H	-0.437572	2.350728	1.326756
H	0.175445	0.641613	-3.370222

C2

C	-1.304621	2.100552	1.757941
C	-2.483962	2.791076	1.481604
C	-3.235552	0.973115	0.083661
C	-2.052699	0.288846	0.367785
C	-1.078900	0.847676	1.197609
H	-0.152349	0.321325	1.411383
Se	-1.807226	-1.387235	-0.490842
C	-0.925889	-2.425844	0.922848
C	0.537744	-2.718069	0.548554
N	1.119934	-1.519024	-0.034932
C	2.145358	-0.698001	0.442245
C	2.071898	0.476999	-0.319902
N	1.000752	0.311520	-1.201749
C	0.462091	-0.900521	-1.035624
C	0.504019	1.300215	-2.160065
C	1.354184	1.354895	-3.421238
H	1.348493	0.387197	-3.928500
H	0.956799	2.108723	-4.104636
H	2.389527	1.616608	-3.186460
H	0.476043	2.264306	-1.642972
H	-0.527004	1.007073	-2.377135
C	2.955312	1.535428	-0.118441
C	3.908914	1.369979	0.876465
C	3.983252	0.191077	1.638737
C	3.107743	-0.866870	1.435143
H	1.106373	-2.996107	1.438485
H	0.602045	-3.537779	-0.170356
H	-1.009152	-1.831026	1.834181
H	-1.499512	-3.346320	1.035775
H	-0.555077	2.537365	2.409554
H	-3.988657	0.525551	-0.557933
C	-3.445248	2.229228	0.645001
H	-4.366113	2.762121	0.433833
H	-2.654261	3.767717	1.922037
H	3.179827	-1.779174	2.017413
H	2.903824	2.442418	-0.711389
H	4.749353	0.102749	2.401049
H	4.618883	2.167105	1.067126

C6

C	0.188866	2.869393	0.110566
C	0.888569	2.848225	-1.095092
C	-0.517270	1.043839	-1.868895
C	-1.211969	1.060358	-0.658244
C	-0.872963	1.994898	0.321621
H	-1.443335	2.057507	1.243810
Se	-2.680618	-0.149825	-0.456547
C	-2.421251	-0.719483	1.390703
C	-1.470462	-1.910773	1.511815
N	-0.182443	-1.604806	0.891794
C	0.755516	-0.717221	1.400888
C	1.698326	-0.492290	0.388712
N	1.283287	-1.254362	-0.699730
C	0.155900	-1.876049	-0.368676

C	1.986297	-1.352465	-1.985667
C	3.228006	-2.227500	-1.882531
H	2.962470	-3.250548	-1.605663
H	3.735749	-2.252552	-2.849197
H	3.927685	-1.837312	-1.138897
H	2.224790	-0.327702	-2.283788
H	1.270385	-1.749059	-2.709789
C	2.774133	0.380455	0.567952
C	2.864354	1.022336	1.792738
C	1.917096	0.813743	2.825691
C	0.884026	-0.052572	2.603343
H	-1.278393	-2.162848	2.558925
H	-1.876178	-2.791347	1.009906
H	-2.070380	0.124500	1.988151
H	-3.408317	-1.005670	1.758880
H	0.451178	3.587857	0.880718
H	-0.800851	0.332856	-2.641768
C	0.522572	1.945651	-2.090459
H	1.037216	1.948301	-3.047149
H	1.699980	3.548415	-1.265459
H	3.500527	0.559020	-0.217197
H	2.023379	1.336244	3.770860
H	3.684760	1.708990	1.972052
H	-0.424046	-2.501689	-1.033027

C7

C	0.166385	2.785198	0.757161
C	0.899609	3.032186	-0.402558
C	-0.467442	1.438915	-1.595932
C	-1.195131	1.185500	-0.431935
C	-0.892711	1.882486	0.738869
H	-1.489395	1.739811	1.635111
Se	-2.655083	-0.046773	-0.544507
C	-2.443387	-1.004200	1.141559
C	-1.476502	-2.184453	1.033196
N	-0.174630	-1.744993	0.536742
C	0.748316	-0.985900	1.248445
C	1.702694	-0.548334	0.320502
N	1.312080	-1.056938	-0.915491
C	0.186050	-1.737760	-0.748251
C	2.039087	-0.875553	-2.178162
C	3.282043	-1.752748	-2.243419
H	3.014814	-2.811165	-2.197058
H	3.806141	-1.570265	-3.184102
H	3.968042	-1.532285	-1.421482
H	2.278821	0.189288	-2.244996
H	1.338442	-1.107060	-2.984139
C	2.778519	0.267726	0.680191
C	2.860064	0.634380	2.017815
C	1.884976	0.171283	2.894898
C	0.815920	-0.630661	2.602338
H	-1.319316	-2.666278	2.002817
H	-1.855427	-2.936118	0.337766
H	-2.130178	-0.303864	1.918134
H	-3.436776	-1.373745	1.403245
H	0.400368	3.316849	1.674044
H	-0.722671	0.914892	-2.514364
C	0.569535	2.370573	-1.582827
H	1.110379	2.586006	-2.499999
H	1.709032	3.754947	-0.390615
H	0.092034	-0.957854	3.342048
H	3.508477	0.607215	-0.047375
H	3.668994	1.267823	2.366091
H	-0.379253	-2.206240	-1.542018

C8

C	0.115418	-2.744819	-0.933661
C	0.849394	-3.083861	0.202137
C	-0.474998	-1.542817	1.507186
C	-1.203717	-1.197862	0.367698
C	-0.923237	-1.822474	-0.848538
H	-1.522175	-1.608194	-1.728910
Se	-2.636808	0.054270	0.570425
C	-2.411706	1.115804	-1.050034
C	-1.421765	2.268052	-0.869039
N	-0.127170	1.773200	-0.406925
C	0.782367	1.045293	-1.169908
C	1.726206	0.529429	-0.271872
N	1.346354	0.961983	0.994401
C	0.231991	1.674831	0.872594
C	2.071657	0.683544	2.240289
C	3.329059	1.533683	2.361596
H	3.079709	2.597111	2.389046
H	3.852348	1.278135	3.285588
H	4.009272	1.358362	1.524080
H	2.294883	-0.386961	2.233424
H	1.375877	0.870392	3.061920
C	2.785857	-0.282040	-0.698353
C	2.783769	-0.505357	-2.047804
C	1.876983	-0.018157	-2.983170
C	0.834676	0.788491	-2.542444
H	-1.258587	2.809535	-1.805532
H	-1.784835	2.979441	-0.124516
H	-2.114729	0.461443	-1.871762
H	-3.398383	1.521057	-1.282701
H	0.333098	-3.219917	-1.884938
H	-0.713061	-1.074820	2.459848
C	0.540995	-2.494027	1.425399
H	1.082577	-2.780510	2.322481
H	1.643040	-3.820996	0.136435
H	0.102201	1.195307	-3.232280
H	3.524135	-0.699689	-0.021356

H	1.978972	-0.256693	-4.036625
H	-0.324841	2.099284	1.696561

C9

C	0.187459	2.835548	0.482920
C	0.898523	2.969678	-0.708705
C	-0.499880	1.281892	-1.722546
C	-1.204302	1.140971	-0.525655
C	-0.876086	1.942168	0.568984
H	-1.455617	1.886320	1.485866
Se	-2.669025	-0.089977	-0.490153
C	-2.410897	-0.891550	1.269409
C	-1.448060	-2.079480	1.242074
N	-0.161859	-1.693685	0.665068
C	0.787850	-0.876466	1.271440
C	1.710252	-0.548004	0.268779
N	1.283765	-1.169560	-0.897497
C	0.158273	-1.817766	-0.625448
C	1.977927	-1.096525	-2.190963
C	3.309336	-1.832611	-2.152280
H	3.166993	-2.891230	-1.922882
H	3.794236	-1.751292	-3.127518
H	3.976760	-1.395743	-1.404466
H	2.107345	-0.032672	-2.408562
H	1.300263	-1.517338	-2.937621
C	2.771255	0.280039	0.573192
C	2.936503	0.785485	1.831901
C	1.993191	0.441765	2.831410
C	0.913482	-0.388559	2.574344
H	-1.259779	-2.468189	2.247269
H	-1.848927	-2.892221	0.632911
H	-2.074679	-0.124587	1.969621
H	-3.396478	-1.232760	1.591968
H	0.442992	3.447631	1.342077
H	-0.774348	0.675552	-2.582959
C	0.540423	2.205184	-1.816237
H	1.064417	2.331508	-2.759375
H	1.713188	3.682902	-0.779220
H	0.202720	-0.644748	3.352601
H	2.127519	0.840818	3.831203
H	3.770253	1.435831	2.075449
H	-0.434856	-2.358479	-1.349877

C10α

C	-0.046741	2.432641	-1.402552
C	0.689840	1.764803	-2.381068
C	-0.653710	-0.212395	-2.039476
C	-1.387105	0.453563	-1.055083
C	-1.096720	1.787847	-0.757369
H	-1.700659	2.326680	-0.032271
Se	-2.839069	-0.457945	-0.215874
C	-2.341243	-0.135169	1.694135
C	-1.308066	-1.115997	2.053776
N	-0.047153	-0.980432	1.423436
C	0.774079	0.149790	1.444552
C	1.794266	-0.084587	0.519222
N	1.563769	-1.347463	-0.019018
C	0.459202	-1.837786	0.527647
C	2.398534	-2.004743	-1.032340
C	3.685339	-2.554619	-0.431696
H	3.468944	-3.316003	0.321418
H	4.287977	-3.010667	-1.220192
H	4.275630	-1.762339	0.035642
H	2.590442	-1.254769	-1.804790
H	1.792232	-2.794920	-1.481940
C	2.794684	0.857147	0.273366
C	2.721935	2.033263	0.999484
C	1.704228	2.258906	1.949539
C	0.714964	1.322911	2.197101
H	-1.575841	-2.136119	2.299827
H	-2.029442	0.906772	1.773375
H	-3.257075	-0.289698	2.263444
H	0.178894	3.466382	-1.160885
H	-0.903634	-1.240583	-2.289647
C	0.374467	0.448553	-2.710379
H	0.916727	-0.062081	-3.501205
H	1.491295	2.279214	-2.901754
H	-0.053635	1.483300	2.945016
H	3.584041	0.680226	-0.449237
H	1.704359	3.188174	2.508532
H	3.474478	2.798381	0.843946
H	-0.000416	-2.781306	0.266365

C10β

C	-0.046630	2.432366	-1.402439
C	0.690376	1.764394	-2.380538
C	-0.653285	-0.212776	-2.039217
C	-1.387099	0.453316	-1.055221
C	-1.096873	1.787654	-0.757613
H	-1.701123	2.326570	-0.032834
Se	-2.839206	-0.458057	-0.216091
C	-2.341490	-0.135017	1.693880
C	-1.308188	-1.115708	2.053637
N	-0.047261	-0.980173	1.423401
C	0.773834	0.150165	1.444237
C	1.794154	-0.084384	0.519085
N	1.563922	-1.347491	-0.018688
C	0.459340	-1.837748	0.527976
C	2.398896	-2.005115	-1.031632
C	3.685657	-2.554622	-0.430586
H	3.469207	-3.315575	0.322945
H	4.288357	-3.011125	-1.218767

H	4.275897	-1.762070	0.036344
H	2.590795	-1.255436	-1.804372
H	1.792691	-2.795494	-1.481004
C	2.794553	0.857345	0.273121
C	2.721630	2.033649	0.998902
C	1.703782	2.259482	1.948765
C	0.714562	1.323485	2.196472
H	-1.575944	-2.135790	2.299860
H	-2.029894	0.906976	1.773124
H	-3.257287	-0.289706	2.263203
H	0.178877	3.466147	-1.160823
H	-0.903058	-1.241022	-2.289316
C	0.375172	0.448086	-2.709775
H	0.917780	-0.062666	-3.500289
H	1.492062	2.278732	-2.900944
H	-0.054109	1.484021	2.944281
H	3.584053	0.680257	-0.449289
H	1.703784	3.188905	2.507502
H	3.474147	2.798773	0.843259
H	-0.000178	-2.781367	0.266892

C11α

C	0.212173	2.432528	-1.296177
C	0.842648	1.804348	-2.369161
C	-0.642012	-0.086395	-2.134464
C	-1.266919	0.545861	-1.059831
C	-0.853483	1.813323	-0.649658
H	-1.368014	2.309647	0.168345
Se	-2.777480	-0.309942	-0.243238
C	-2.472396	0.078161	1.562495
C	-1.385234	-0.594104	2.352368
N	-0.145519	-0.741099	1.592365
C	0.870566	0.203056	1.482933
C	1.737332	-0.264412	0.490460
N	1.207877	-1.468528	0.035285
C	0.086520	-1.707611	0.703266
C	1.800809	-2.319438	-1.003652
C	3.011846	-3.082474	-0.484281
H	2.729072	-3.754418	0.329543
H	3.438648	-3.680232	-1.292618
H	3.783964	-2.400914	-0.118366
H	2.054286	-1.656542	-1.835849
H	1.014601	-2.995666	-1.348104
C	2.871244	0.452038	0.105373
C	3.089313	1.652834	0.758244
C	2.214113	2.124302	1.758439
C	1.089832	1.413169	2.141644
H	-1.150096	-0.038776	3.264998
H	-1.691637	-1.601503	2.665218
H	-3.356068	0.358586	2.125674
H	0.536923	3.416347	-0.972382
H	-0.985159	-1.065245	-2.461737
C	0.408268	0.550367	-2.794062
H	0.876241	0.070641	-3.648890
H	1.659754	2.299437	-2.883954
H	0.413277	1.785190	2.903291
H	3.540204	0.093854	-0.669688
H	2.429591	3.071366	2.240858
H	3.956525	2.248496	0.495521
H	-0.572026	-2.548199	0.531048

C11β

C	0.212505	2.431420	-1.297906
C	0.842840	1.802523	-2.370591
C	-0.642012	-0.087934	-2.134640
C	-1.266738	0.545032	-1.060404
C	-0.853177	1.812738	-0.650993
H	-1.367630	2.309573	0.166739
Se	-2.777311	-0.309977	-0.242981
C	-2.472360	0.080115	1.562398
C	-1.385387	-0.591655	2.353058
N	-0.145557	-0.739697	1.593319
C	0.870597	0.204281	1.482770
C	1.737088	-0.264169	0.490514
N	1.207366	-1.468621	0.036483
C	0.086128	-1.706983	0.704934
C	1.800003	-2.320419	-1.001915
C	3.010827	-3.083457	-0.482062
H	2.727909	-3.754802	0.332208
H	3.437463	-3.681860	-1.290013
H	3.783126	-2.401860	-0.116603
H	2.053664	-1.658177	-1.834578
H	1.013584	-2.996666	-1.345852
C	2.871197	0.451650	0.104802
C	3.089721	1.652829	0.756808
C	2.214816	2.125269	1.756802
C	1.090363	1.414765	2.140647
H	-1.150261	-0.035509	3.265180
H	-1.692019	-1.598718	2.666684
H	-3.355699	0.362203	2.125264
H	0.537422	3.415389	-0.974745
H	-0.985297	-1.066908	-2.461394
C	0.408315	0.548365	-2.794690
H	0.876157	0.068107	-3.649293
H	1.659963	2.297206	-2.885746
H	0.414032	1.787484	2.902148
H	3.539920	0.092768	-0.670141
H	2.430674	3.072596	2.238533
H	3.957099	2.248030	0.493580
H	-0.572554	-2.547635	0.533575

C13

C	0.125433	2.927042	-0.075507
C	0.869005	2.821548	-1.252448
C	-0.469475	0.974486	-1.870129
C	-1.232582	1.035377	-0.728710
C	-0.934807	2.061089	0.179282
H	-1.536400	2.191034	1.075271
Se	-2.683481	-0.191696	-0.502988
C	-2.431606	-0.660457	1.374378
C	-1.465170	-1.831123	1.561511
N	-0.176149	-1.544139	0.937835
C	0.767420	-0.634866	1.407967
C	1.700394	-0.465341	0.380083
N	1.279888	-1.271810	-0.673756
C	0.153968	-1.874234	-0.311676
C	1.971448	-1.426138	-1.959468
C	3.178893	-2.346006	-1.841239
H	2.875592	-3.351894	-1.541042
H	3.682347	-2.412140	-2.808220
H	3.894659	-1.965924	-1.107571
H	2.250832	-0.419188	-2.280340
H	1.235609	-1.804916	-2.673075
C	2.784598	0.403404	0.506837
C	2.883390	1.091933	1.704332
C	1.943054	0.920525	2.740139
C	0.868721	0.055299	2.616185
H	-1.285533	-2.039002	2.620466
H	-1.859041	-2.738477	1.099100
H	-2.098078	0.222511	1.923041
H	-3.417964	-0.939970	1.749717
H	0.354326	3.711896	0.637838
C	0.561326	1.829965	-2.190055
H	1.101647	1.756316	-3.129553
H	1.672973	3.523140	-1.453039
H	0.148254	-0.076199	3.416561
H	3.507153	0.538912	-0.290729
H	2.067417	1.479974	3.660754
H	3.707858	1.780595	1.852068
H	-0.433521	-2.522359	-0.947106

C14

C	0.126344	2.764131	-0.901694
C	0.877182	2.326169	-1.999222
C	-0.518634	0.319259	-2.116267
C	-1.244965	0.768445	-1.004479
C	-0.934975	1.999720	-0.421888
H	-1.533960	2.378918	0.400573
Se	-2.697437	-0.315536	-0.388587
C	-2.397825	-0.226439	1.537598
C	-1.420432	-1.292762	2.034034
N	-0.141823	-1.194742	1.334503
C	0.799278	-0.183062	1.502168
C	1.721899	-0.323364	0.460558
N	1.300706	-1.411856	-0.298178
C	0.182404	-1.883620	0.239617
C	1.980368	-1.931148	-1.492341
C	3.246146	-2.696853	-1.132450
H	3.012842	-3.567403	-0.514782
H	3.732475	-3.042925	-2.047082
H	3.952544	-2.065007	-0.588083
H	2.183450	-1.066602	-2.131758
H	1.261141	-2.565957	-2.015788
C	2.799535	0.550361	0.310218
C	2.903484	1.564298	1.247376
C	1.974602	1.703459	2.298616
C	0.906469	0.835255	2.449906
H	-1.223975	-1.188218	3.105288
H	-1.815252	-2.295284	1.857100
H	-2.059329	0.777245	1.801915
H	-3.373498	-0.386540	2.000604
H	0.356392	3.717012	-0.434512
H	-0.764712	-0.625386	-2.596049
C	0.507155	1.124217	-2.556080
H	1.695275	2.920369	-2.393645
H	0.194661	0.943976	3.261363
H	3.510876	0.448397	-0.502253
H	2.103693	2.511602	3.010132
H	3.722890	2.270690	1.173454
H	-0.403039	-2.698879	-0.162803

C15

C	0.107083	2.386826	-1.659596
C	0.768113	1.613545	-2.587905
C	-0.550788	-0.298453	-2.135688
C	-1.244857	0.436787	-1.171186
C	-0.938767	1.783551	-0.956981
H	-1.518987	2.375278	-0.254236
Se	-2.678329	-0.436602	-0.251170
C	-2.382775	0.214775	1.563822
C	-1.383475	-0.638331	2.346283
N	-0.110132	-0.731061	1.636763
C	0.810840	0.301490	1.483446
C	1.728120	-0.125778	0.518163
N	1.323188	-1.397713	0.122537
C	0.219331	-1.708254	0.791559
C	2.019452	-2.247819	-0.850914
C	3.276842	-2.868783	-0.257588
H	3.030487	-3.515946	0.587599
H	3.778700	-3.470725	-1.018291
H	3.974227	-2.100236	0.085475
H	2.240501	-1.612197	-1.712793
H	1.306600	-3.009777	-1.175740

C	2.785418	0.682261	0.098690
C	2.875130	1.931364	0.689512
C	1.951952	2.360637	1.664539
C	0.903605	1.558024	2.082723
H	-1.184735	-0.216936	3.336234
H	-1.759588	-1.654787	2.478849
H	-2.068911	1.259624	1.525423
H	-3.356086	0.174476	2.056851
H	0.359843	3.428793	-1.490061
H	-0.817400	-1.338084	-2.315224
C	0.472870	0.301737	-2.878462
H	0.994647	-0.250927	-3.654681
H	0.196106	1.890991	2.834798
H	3.492371	0.356620	-0.656741
H	2.069178	3.346551	2.100736
H	3.677551	2.598767	0.395476
H	-0.351295	-2.617056	0.658499

C16

C	0.206657	-1.835249	-2.156967
C	0.915641	-2.746644	-1.406506
C	-0.523484	-2.130776	0.443103
C	-1.207048	-1.218747	-0.365708
C	-0.855009	-1.082873	-1.716069
H	-1.405642	-0.429637	-2.386930
Se	-2.677618	-0.270596	0.410516
C	-2.427697	1.491493	-0.387821
C	-1.466286	2.371883	0.412751
N	-0.175555	1.710376	0.583945
C	0.765983	1.498519	-0.419478
C	1.696541	0.591056	0.096023
N	1.279109	0.286294	1.388830
C	0.155977	0.951377	1.629441
C	1.978566	-0.607338	2.319946
C	3.219599	0.051355	2.906934
H	2.953159	0.940298	3.483651
H	3.724867	-0.652567	3.571838
H	3.921521	0.344771	2.122115
H	2.218756	-1.512934	1.755881
H	1.260927	-0.882830	3.096649
C	2.773185	0.130306	-0.662210
C	2.865512	0.615699	-1.955719
C	1.927813	1.531214	-2.474757
C	0.862628	1.993845	-1.720170
H	-1.288853	3.328779	-0.086977
H	-1.864478	2.577211	1.408537
H	-2.096068	1.377334	-1.421532
H	-3.416216	1.954778	-0.401796
H	-0.819519	-2.247309	1.482784
C	0.518716	-2.901333	-0.074838
H	1.017327	-3.629021	0.560062
H	1.728455	-3.331637	-1.824871
H	0.143782	2.699611	-2.122879
H	3.492022	-0.578857	-0.266104
H	2.046344	1.883594	-3.493381
H	3.681141	0.284040	-2.588529
H	-0.427149	0.875248	2.536768

C17

C	0.316140	0.782824	2.645800
C	0.884635	2.010714	2.286525
C	-0.665169	2.207435	0.428744
C	-1.248189	0.981443	0.776117
C	-0.729571	0.338042	1.873770
Se	-2.721464	0.303709	-0.234974
C	-2.385880	-1.599837	0.019728
C	-1.440010	-2.174058	-1.038622
N	-0.180671	-1.433211	-1.077847
C	0.852774	-1.532405	-0.149668
C	1.708104	-0.452867	-0.392263
N	1.154360	0.262274	-1.450745
C	0.030613	-0.341686	-1.816553
C	1.728921	1.471481	-2.053464
C	2.926633	1.147270	-2.935887
H	2.633402	0.504243	-3.769176
H	3.339485	2.072791	-3.343108
H	3.712885	0.642367	-2.368835
H	1.991441	2.133378	-1.223236
H	0.929016	1.954686	-2.619761
C	2.849019	-0.228840	0.377995
C	3.086046	-1.131519	1.401168
C	2.221677	-2.216998	1.648709
C	1.089980	-2.441042	0.881783
H	-1.203409	-3.222787	-0.837705
H	-1.887432	-2.113802	-2.032932
H	-1.997804	-1.740795	1.031790
H	-3.354877	-2.097071	-0.050960
H	0.680239	0.220039	3.499707
H	-1.046125	2.763134	-0.425848
C	0.389244	2.714929	1.188281
H	0.818881	3.676862	0.925626
H	1.703568	2.417792	2.871544
H	0.425981	-3.275969	1.078287
H	3.509819	0.611287	0.193503
H	2.451449	-2.897079	2.461584
H	3.960310	-1.001894	2.029489
H	-0.642671	0.013981	-2.583678

C18α

C	0.672244	0.839195	2.547494
C	1.378042	1.912597	2.004240
C	-0.305093	2.139381	0.288090

C	-1.005135	1.058941	0.826050
C	-0.526912	0.426396	1.975412
H	-1.092676	-0.377278	2.437716
Se	-2.658987	0.570199	-0.003984
C	-2.531182	-1.374946	0.055768
C	-1.774459	-1.957368	-1.139511
N	-0.434770	-1.383940	-1.234676
C	0.618755	-1.646097	-0.367692
C	1.609613	-0.688614	-0.618735
N	1.108930	0.135839	-1.649110
C	-0.122251	-0.310016	-1.956905
C	1.684026	1.248265	-2.247596
C	3.055791	1.712139	-1.939697
H	3.809867	0.939823	-2.138549
H	3.286937	2.571591	-2.569978
H	3.154634	2.026915	-0.892762
H	1.062335	1.714560	-2.999247
C	2.796818	-0.680464	0.117520
C	2.932411	-1.650991	1.097470
C	1.931703	-2.607858	1.344626
C	0.756040	-2.625930	0.614242
H	-1.668651	-3.043256	-1.058018
H	-2.292951	-1.735482	-2.074425
H	-2.077321	-1.679325	1.000828
H	-3.559745	-1.741032	0.043599
H	1.042495	0.336926	3.435595
H	-0.689670	2.644710	-0.594758
C	0.878722	2.571746	0.883835
H	1.402220	3.430807	0.473882
H	2.300374	2.247257	2.468364
H	-0.017304	-3.364529	0.796601
H	3.581286	0.043873	-0.048036
H	2.087427	-3.349337	2.120446
H	3.840632	-1.672654	1.689411
H	-0.778226	0.158133	-2.676377

C18β

C	0.873150	0.657589	-2.659459
C	1.387831	-0.632904	-2.779677
C	-0.451140	-1.479280	-1.466241
C	-0.956891	-0.185981	-1.332031
C	-0.306282	0.880126	-1.955624
H	-0.723502	1.881934	-1.910145
Se	-2.592672	0.038955	-0.364729
C	-2.173428	1.645184	0.659812
C	-1.457133	1.341212	1.977233
N	-0.238161	0.572791	1.743443
C	0.927684	1.062513	1.162504
C	1.722881	-0.043302	0.855629
N	1.003528	-1.182050	1.264704
C	-0.171816	-0.760857	1.763957
C	1.408878	-2.494160	1.123960
C	0.534437	-3.594166	1.590386
H	-0.442587	-3.581048	1.087032
H	1.004001	-4.555737	1.383741
H	0.349777	-3.537787	2.672682
H	2.396991	-2.631834	0.713143
C	2.966129	0.089798	0.244506
C	3.372286	1.382331	-0.049063
C	2.571325	2.498183	0.258533
C	1.335786	2.363342	0.871678
H	-1.178191	2.259752	2.502332
H	-2.094137	0.751505	2.639609
H	-1.588481	2.325324	0.037647
H	-3.131544	2.123424	0.872704
H	1.377795	1.492181	-3.135755
H	-0.976587	-2.311424	-1.003286
C	0.716231	-1.702836	-2.194108
H	1.093544	-2.715055	-2.306303
H	2.297796	-0.804210	-3.345735
H	0.722165	3.225171	1.111450
H	3.585487	-0.765210	-0.001675
H	2.933667	3.490210	0.012356
H	4.332841	1.538411	-0.527152
H	-0.965329	-1.406026	2.109162

C19α

C	0.289874	2.058290	1.959297
C	0.965223	2.843169	1.026124
C	-0.512741	2.038729	-0.706314
C	-1.183957	1.247914	0.227892
C	-0.796539	1.281462	1.568157
H	-1.347271	0.717551	2.315341
Se	-2.687985	0.232759	-0.379756
C	-2.415454	-1.426853	0.607517
C	-1.490559	-2.401963	-0.122145
N	-0.205988	-1.774943	-0.422521
C	0.785106	-1.466524	0.505681
C	1.708782	-0.651595	-0.156030
N	1.235912	-0.494823	-1.456343
C	0.088409	-1.154254	-1.564763
C	1.894393	0.296028	-2.506988
C	3.256264	-0.223738	-2.805129
H	3.413470	-1.288572	-2.922499
H	4.020517	0.459364	-3.149194
H	1.922680	1.332660	-2.156589
H	1.225715	0.264601	-3.379071
C	2.836550	-0.141598	0.487675
C	2.984578	-0.479421	1.822330
C	2.052921	-1.300120	2.490035
C	0.938171	-1.813452	1.848285
H	-1.293918	-3.295181	0.478273

H	-1.930000	-2.719367	-1.069989
H	-2.040777	-1.195612	1.606326
H	-3.403852	-1.877727	0.715201
H	0.590897	2.070756	3.001984
H	-0.833042	2.031686	-1.745924
C	0.550292	2.845719	-0.303174
H	1.045615	3.485173	-1.028575
H	1.796208	3.466788	1.339244
H	0.226021	-2.449002	2.363928
H	3.554507	0.483614	-0.031472
H	2.218301	-1.540199	3.534590
H	3.843527	-0.107478	2.369801
H	-0.534429	-1.175246	-2.448303

C19β

C	0.883681	0.243161	-2.697772
C	1.359083	-1.067000	-2.672423
C	-0.491932	-1.699899	-1.257431
C	-0.962577	-0.385816	-1.275814
C	-0.285416	0.579823	-2.021744
H	-0.671333	1.592440	-2.093407
Se	-2.592908	-0.016397	-0.344929
C	-2.180732	1.726554	0.427059
C	-1.458016	1.621243	1.770845
N	-0.238231	0.828306	1.644404
C	0.924009	1.227285	0.990859
C	1.717258	0.083080	0.862053
N	1.002845	-0.961322	1.441445
C	-0.158941	-0.484008	1.870992
C	1.480932	-2.360059	1.529425
C	0.434921	-3.291434	2.029433
H	-0.372431	-3.603109	1.378821
H	0.534212	-3.762868	2.997098
H	2.361492	-2.355934	2.176973
H	1.794374	-2.620959	0.511342
C	2.963333	0.116191	0.236844
C	3.368397	1.344595	-0.258568
C	2.567981	2.497476	-0.131515
C	1.333549	2.465624	0.496514
H	-1.178632	2.607020	2.154583
H	-2.090502	1.133252	2.515166
H	-1.603912	2.312904	-0.290568
H	-3.142101	2.223621	0.570771
H	1.409582	1.002810	-3.267364
H	-1.041197	-2.456698	-0.702252
C	0.658887	-2.041973	-1.965858
H	0.999254	-3.073731	-1.973788
H	2.257816	-1.330525	-3.220525
H	0.722988	3.356728	0.597491
H	3.579543	-0.770572	0.136857
H	2.932527	3.436943	-0.532252
H	4.328888	1.424021	-0.755587
H	-0.940508	-1.075797	2.325869

C19γ

C	0.883240	0.240075	-2.698166
C	1.358455	-1.070128	-2.671525
C	-0.492410	-1.701284	-1.255554
C	-0.962878	-0.387156	-1.275257
C	-0.285709	0.577606	-2.022317
H	-0.671510	1.590195	-2.094985
Se	-2.593052	-0.016548	-0.344560
C	-2.180562	1.727138	0.425610
C	-1.457675	1.623100	1.769403
N	-0.237934	0.829995	1.643584
C	0.924272	1.228332	0.989566
C	1.717473	0.083984	0.861747
N	1.003090	-0.959861	1.442191
C	-0.158662	-0.482120	1.871380
C	1.481235	-2.358505	1.531386
C	0.435298	-3.289480	2.032280
H	-0.370797	-3.603550	1.381262
H	0.533614	-3.758458	3.001244
H	2.361838	-2.353701	2.178896
H	1.794620	-2.620311	0.513532
C	2.963494	0.116463	0.236397
C	3.368560	1.344394	-0.260187
C	2.568201	2.497424	-0.134118
C	1.333821	2.466199	0.494049
H	-1.178206	2.609242	2.152145
H	-2.090083	1.135862	2.514285
H	-1.603749	2.312673	-0.292688
H	-3.141841	2.224482	0.568957
H	1.409160	0.999044	-3.268646
H	-1.041685	-2.457406	-0.699461
C	0.658235	-2.044262	-1.963827
H	0.998462	-3.076074	-1.970738
H	2.257051	-1.334354	-3.219513
H	0.723300	3.357417	0.594253
H	3.579661	-0.770416	0.137179
H	2.932753	3.436508	-0.535746
H	4.329016	1.423330	-0.757351
H	-0.940253	-1.073476	2.326783

Table S6. Coordinates and imaginary frequencies (Nimag, cm^{-1}) of the transition states of HAT and RAF (from C2 site). Level of theory: M06-2X/6-31G(d).

C10α

Nimag= -1552.6034

C	0.172739	-2.806132	-0.470732
C	0.857005	-2.947664	0.735991
C	-0.570273	-1.275307	1.734227
C	-1.251070	-1.130500	0.523918
C	-0.893077	-1.917791	-0.572251
H	-1.451625	-1.853607	-1.501526
Se	-2.731468	0.076967	0.469300
C	-2.425144	0.928418	-1.265386
C	-1.470044	2.095578	-1.132316
N	-0.177691	1.706795	-0.609480
C	0.763260	0.899667	-1.251564
C	1.697749	0.536039	-0.276882
N	1.290114	1.136421	0.912197
C	0.167907	1.801646	0.678308
C	1.994710	1.043409	2.196690
C	3.237471	1.923051	2.220973
H	2.972075	2.975896	2.098764
H	3.747315	1.805025	3.179622
H	3.934710	1.646413	1.426018
H	2.233051	-0.014096	2.340111
H	1.281107	1.329663	2.973256
C	2.777136	-0.298925	-0.566041
C	2.868327	-0.750493	-1.871781
C	1.930991	-0.374294	-2.855261
C	0.862600	0.461399	-2.573307
H	-1.313168	2.501052	-2.261067
H	-1.868454	2.918669	-0.537057
H	-2.059442	0.189395	-1.980394
H	-3.396137	1.287835	-1.609288
H	0.451559	-3.407898	-1.329796
H	-0.868727	-0.679470	2.593832
C	0.473326	-2.193308	1.842053
H	0.976968	-2.325376	2.795446
H	1.671801	-3.659737	0.818301
H	3.499132	-0.586773	0.190490
H	2.056435	-0.743827	-3.867174
H	3.687270	-1.405303	-2.148592
H	-0.413924	2.321507	1.427100
H	0.156213	0.783625	-3.331046
O	-1.197274	2.688568	-3.564136
H	-0.805412	3.584226	-3.609080

C10β

Nimag= -1654.7625

C	0.088057	-2.847560	-0.441622
C	0.797462	-2.967058	0.752231
C	-0.530769	-1.191571	1.707305
C	-1.235496	-1.066238	0.508212
C	-0.940777	-1.917337	-0.557468
H	-1.518278	-1.872970	-1.476273
Se	-2.637333	0.235726	0.447447
C	-2.395935	0.918067	-1.364225
C	-1.390622	2.053496	-1.416553
N	-0.121773	1.708203	-0.813802
C	0.832311	0.850992	-1.364355
C	1.719204	0.536932	-0.330604
N	1.265092	1.210194	0.802075
C	0.161152	1.872215	0.484409
C	1.918289	1.195985	2.116407
C	3.171335	2.060917	2.131679
H	2.925326	3.105647	1.927068
H	3.640805	2.003680	3.116161
H	3.896362	1.722971	1.386735
H	2.136414	0.148054	2.340663
H	1.178665	1.543162	2.841836
C	2.794206	-0.329686	-0.524613
C	2.933012	-0.863461	-1.795636
C	2.037991	-0.546322	-2.835875
C	0.969964	0.315754	-2.643811
H	-1.241904	2.502249	-2.401654
H	-1.867362	2.904877	-0.681099
H	-2.097572	0.099995	-2.026382
H	-3.373227	1.279114	-1.690051
H	0.316859	-3.499054	-1.279036
H	-0.777191	-0.542597	2.544931
C	0.473982	-2.149292	1.832130
H	0.997006	-2.259975	2.777824
H	1.583898	-3.708884	0.845936
H	3.479124	-0.581816	0.277808
H	2.191061	-0.988080	-3.814354
H	3.752851	-1.544087	-1.996699
H	-0.447053	2.466709	1.154843
H	0.281225	0.557872	-3.446355
O	-2.301075	3.552537	0.347490
H	-2.302805	4.468606	-0.000078

C11α

Nimag= -1121.8849

C	0.268003	-2.747893	-0.413514
C	0.911795	-2.909891	0.811840
C	-0.583147	-1.295330	1.804891
C	-1.223463	-1.137230	0.574625
C	-0.811551	-1.876543	-0.535498
H	-1.316753	-1.773387	-1.491847

Se	-2.747412	0.022108	0.522865
C	-2.541033	0.902009	-1.167129
C	-1.458822	1.971598	-1.282750
N	-0.187808	1.555872	-0.701931
C	0.785925	0.761887	-1.303964
C	1.728402	0.478106	-0.310204
N	1.284184	1.102434	0.851604
C	0.140697	1.717719	0.580471
C	1.977223	1.082971	2.145021
C	3.187963	2.006421	2.148633
H	2.887008	3.043912	1.984616
H	3.691895	1.941992	3.115485
H	3.902690	1.726018	1.370695
H	2.250681	0.040720	2.331580
H	1.245319	1.372433	2.903135
C	2.845172	-0.320294	-0.559364
C	2.964828	-0.819620	-1.844664
C	2.010412	-0.537030	-2.843714
C	0.902853	0.257524	-2.599852
H	-1.284180	2.217958	-2.333674
H	-1.769961	2.882639	-0.764779
H	-2.334726	0.094125	-2.007035
H	-3.513854	1.339681	-1.403141
H	0.594742	-3.313615	-1.280291
H	-0.923230	-0.729792	2.669445
C	0.478094	-2.190941	1.923605
H	0.955474	-2.333307	2.888769
H	1.738667	-3.606835	0.903594
H	3.572584	-0.546875	0.212649
H	2.147177	-0.958399	-3.833546
H	3.813870	-1.447698	-2.091158
H	-0.462676	2.252443	1.301404
H	0.149184	0.442189	-3.357259
O	-2.040195	-0.387343	-3.271350
H	-2.942634	-0.527327	-3.623265

C11β

Nimag= -1633.4245

C	0.104167	-2.861605	-0.490267
C	0.821418	-3.012170	0.695569
C	-0.527657	-1.292150	1.721703
C	-1.239526	-1.139701	0.531195
C	-0.938994	-1.943690	-0.568117
H	-1.520831	-1.868725	-1.482071
Se	-2.664022	0.146515	0.510517
C	-2.375675	0.928237	-1.224691
C	-1.431400	2.122682	-1.234139
N	-0.137866	1.742958	-0.666341
C	0.792466	0.903880	-1.274769
C	1.704290	0.534675	-0.281077
N	1.285881	1.158195	0.891489
C	0.179587	1.842550	0.625222
C	1.969757	1.079562	2.187905
C	3.229401	1.934782	2.212949
H	2.987059	2.989435	2.062001
H	3.721360	1.829155	3.182326
H	3.933876	1.626315	1.436247
H	2.184838	0.020950	2.358860
H	1.250684	1.397636	2.946837
C	2.768715	-0.328264	-0.543011
C	2.869281	-0.803010	-1.840028
C	1.949810	-0.430850	-2.841304
C	0.895083	0.428728	-2.582216
H	-1.270635	2.494906	-2.248942
H	-1.836879	2.940054	-0.633752
H	-2.139507	0.182222	-1.984522
H	-3.445322	1.444487	-1.521653
H	0.339787	-3.478079	-1.351886
H	-0.779937	-0.679432	2.584268
C	0.493101	-2.238055	1.805569
H	1.023192	-2.374649	2.743871
H	1.619246	-3.744963	0.758984
H	3.473766	-0.620032	0.227862
H	2.075539	-0.826228	-3.843244
H	3.678633	-1.478262	-2.094712
H	-0.400640	2.390494	1.354827
H	0.191126	0.716619	-3.355860
O	-4.241973	2.225251	-2.156516
H	-4.680230	1.594722	-2.762741

C18α

Nimag= -1676.385

C	0.168332	-2.807062	-0.393364
C	0.840347	-2.907878	0.823154
C	-0.642058	-1.258526	1.773246
C	-1.301860	-1.142811	0.548380
C	-0.915651	-1.943293	-0.526737
H	-1.461435	-1.907377	-1.465176
Se	-2.777726	0.070379	0.444574
C	-2.438962	0.868602	-1.301351
C	-1.453876	2.034755	-1.234090
N	-0.184825	1.618894	-0.638705
C	0.781736	0.826762	-1.253930
C	1.717749	0.499813	-0.268318
N	1.277806	1.109164	0.908643
C	0.130457	1.736984	0.647470
C	1.985679	1.146550	2.172441
C	3.202983	2.046248	2.148500
H	2.910088	3.082005	1.948400
H	3.706335	2.017370	3.117820
H	3.910464	1.730274	1.377722
H	2.374909	0.000519	2.380456

H	1.273679	1.364477	2.971138
C	2.813266	-0.323237	-0.531555
C	2.923077	-0.790242	-1.830950
C	1.985314	-0.454207	-2.828129
C	0.895420	0.358097	-2.562701
H	-1.234263	2.436057	-2.228092
H	-1.853068	2.844497	-0.619968
H	-2.096357	0.096795	-1.992943
H	-3.404072	1.234994	-1.656859
H	0.474200	-3.415660	-1.238581
H	-0.965924	-0.652687	2.616792
C	0.418041	-2.150476	1.913396
H	0.929627	-2.239300	2.865224
H	1.681659	-3.584893	0.928928
H	3.507347	-0.608008	0.251858
H	2.120043	-0.845909	-3.830393
H	3.752929	-1.438885	-2.088953
H	-0.469374	2.262664	1.377692
H	0.171418	0.610283	-3.330201
O	3.161330	-1.025027	2.541917
H	3.735888	-0.708834	3.269136

C18β

Nimag= -1543.3458

C	0.109257	-2.865943	-0.397205
C	0.837902	-2.983705	0.785641
C	-0.480233	-1.210702	1.767139
C	-1.202254	-1.090235	0.578195
C	-0.923399	-1.938224	-0.494836
H	-1.518462	-1.892156	-1.402390
Se	-2.627194	0.186301	0.531642
C	-2.381415	0.927940	-1.255532
C	-1.389388	2.092523	-1.275093
N	-0.101070	1.681782	-0.721544
C	0.804376	0.821154	-1.338448
C	1.737391	0.459326	-0.362416
N	1.355545	1.116150	0.807421
C	0.244372	1.810015	0.557647
C	2.035419	1.034193	2.087478
C	3.050011	2.130330	2.313458
H	2.576362	3.114708	2.268808
H	3.523939	2.009169	3.290000
H	3.831427	2.088338	1.546505
H	2.407609	0.015242	2.214827
H	1.126865	1.142407	2.886926
C	2.792526	-0.408802	-0.637570
C	2.856887	-0.906536	-1.928946
C	1.914394	-0.546175	-2.911821
C	0.872806	0.326236	-2.640194
H	-1.215552	2.457839	-2.291605
H	-1.754492	2.924939	-0.670404
H	-2.075294	0.134723	-1.940353
H	-3.363312	1.285558	-1.571461
H	0.326104	-3.516532	-1.238539
H	-0.706404	-0.554307	2.604415
C	0.530504	-2.166095	1.870509
H	1.069461	-2.276709	2.807580
H	1.626437	-3.725033	0.866690
H	3.519743	-0.687993	0.117125
H	2.011163	-0.959326	-3.909736
H	3.656384	-1.589975	-2.192640
H	-0.313931	2.350662	1.310968
H	0.152225	0.607088	-3.401009
O	0.037028	1.627627	3.443804
H	0.406653	1.989075	4.275318

C19α

Nimag= -1536.2888

C	0.251595	-2.808761	-0.422254
C	0.982350	-2.901457	0.761254
C	-0.450929	-1.238026	1.765444
C	-1.176109	-1.139064	0.576797
C	-0.838331	-1.947999	-0.509043
H	-1.428606	-1.922827	-1.420276
Se	-2.676150	0.048620	0.547429
C	-2.493377	0.812829	-1.237736
C	-1.557133	2.021941	-1.270390
N	-0.241135	1.675284	-0.740286
C	0.700661	0.868648	-1.373427
C	1.675578	0.565276	-0.416835
N	1.277412	1.196962	0.761827
C	0.127145	1.821258	0.531224
C	1.973803	1.163385	2.050741
C	3.083033	2.187589	2.145154
H	2.815036	3.177412	1.770611
H	3.498588	2.237849	3.152310
H	3.994898	1.786508	1.426919
H	2.355824	0.147032	2.181571
H	1.215994	1.330861	2.823673
C	2.765598	-0.254824	-0.710022
C	2.820813	-0.754853	-2.000390
C	1.838437	-0.450921	-2.964030
C	0.760784	0.369026	-2.674310
H	-1.418822	2.395200	-2.289427
H	-1.952975	2.836515	-0.660425
H	-2.165404	0.036712	-1.931675
H	-3.497096	1.125084	-1.532631
H	0.514914	-3.425919	-1.275321
H	-0.733874	-0.626624	2.619578
C	0.618500	-2.127167	1.860044
H	1.159816	-2.219724	2.797324
H	1.818866	-3.589011	0.831722

H	3.535743	-0.465777	0.022936
H	1.933604	-0.865397	-3.961707
H	3.649905	-1.395111	-2.280610
H	-0.450978	2.350668	1.276000
H	0.007884	0.607038	-3.418250
O	4.928185	1.027515	0.972071
H	5.677345	1.310367	1.533233

C19β

Nimag= -1651.3867

C	0.231754	-2.816051	-0.349611
C	0.949551	-2.891940	0.843118
C	-0.463041	-1.179075	1.792038
C	-1.176102	-1.098357	0.594641
C	-0.842447	-1.939305	-0.467977
H	-1.426028	-1.928484	-1.383891
Se	-2.660621	0.107207	0.521783
C	-2.444322	0.831694	-1.276303
C	-1.502027	2.036068	-1.318460
N	-0.199385	1.696694	-0.751303
C	0.753352	0.871944	-1.343774
C	1.696794	0.581813	-0.353476
N	1.275900	1.238840	0.799476
C	0.137626	1.866006	0.527787
C	1.979668	1.225908	2.088517
C	3.208362	2.108917	2.079015
H	2.994450	3.177486	2.048352
H	3.707623	1.850597	3.182962
H	3.971892	1.820755	1.354531
H	2.239319	0.184432	2.296445
H	1.271771	1.545793	2.855981
C	2.790212	-0.249079	-0.597883
C	2.887862	-0.774226	-1.875486
C	1.937855	-0.480465	-2.874391
C	0.853936	0.347314	-2.632483
H	-1.340220	2.384443	-2.342823
H	-1.906485	2.866945	-0.736797
H	-2.106753	0.039476	-1.947162
H	-3.441023	1.141527	-1.596385
H	0.490577	-3.460550	-1.183708
H	-0.740910	-0.541284	2.628251
C	0.589768	-2.084153	1.919028
H	1.120698	-2.161735	2.863586
H	1.771566	-3.593783	0.939688
H	3.517129	-0.482911	0.172625
H	2.061911	-0.913532	-3.860831
H	3.718105	-1.428911	-2.116201
H	-0.452835	2.417733	1.246025
H	0.125419	0.571821	-3.404361
O	3.730117	1.485443	4.395271
H	3.902009	2.342761	4.833230

C19γ

Nimag= -1473.8275

C	0.102012	-2.823264	-0.361634
C	0.814524	-2.940749	0.830920
C	-0.494923	-1.147462	1.779346
C	-1.200777	-1.023191	0.581390
C	-0.917220	-1.883249	-0.480626
H	-1.497457	-1.835404	-1.397537
Se	-2.603464	0.275924	0.505341
C	-2.328065	0.998358	-1.285665
C	-1.299481	2.130101	-1.314686
N	-0.024928	1.683372	-0.760150
C	0.853611	0.790135	-1.368103
C	1.786360	0.426359	-0.392142
N	1.427531	1.107743	0.768100
C	0.340177	1.829422	0.512416
C	2.140526	1.056819	2.045650
C	3.228704	2.104956	2.101736
H	2.614827	3.151951	1.975497
H	3.739597	2.139125	3.064211
H	3.926750	2.077331	1.262445
H	2.528677	0.038607	2.152136
H	1.406575	1.216806	2.838484
C	2.816827	-0.473773	-0.660089
C	2.859695	-0.996209	-1.942497
C	1.916638	-0.633565	-2.924110
C	0.897467	0.267035	-2.659981
H	-1.117326	2.480830	-2.334922
H	-1.636605	2.979361	-0.717193
H	-2.048478	0.189025	-1.962926
H	-3.298996	1.382803	-1.604121
H	0.322371	-3.482171	-1.195508
H	-0.736128	-0.493264	2.614220
C	0.503466	-2.112025	1.906214
H	1.028471	-2.221317	2.850983
H	1.593522	-3.690169	0.927362
H	3.543591	-0.757241	0.093562
H	1.995050	-1.067715	-3.914722
H	3.641793	-1.701809	-2.200077
H	-0.161464	2.456947	1.235183
H	0.176037	0.549029	-3.419638
O	1.565550	3.936547	2.016557
H	1.871391	4.573872	2.692239

C2

Nimag= -636.1784

C	-0.018804	2.923221	-0.469113
C	0.695744	2.735979	-1.651941
C	-0.629139	0.774315	-2.130286
C	-1.336045	0.955218	-0.939710

C	-1.046148	2.050490	-0.123974
H	-1.627531	2.238866	0.773754
Se	-2.742089	-0.279674	-0.540864
C	-2.485193	-0.487286	1.382239
C	-1.402088	-1.506601	1.739001
N	-0.135483	-1.183425	1.089866
C	0.762735	-0.204244	1.451165
C	1.715487	-0.133222	0.416343
N	1.346108	-1.064029	-0.528844
C	0.243520	-1.705083	-0.098424
C	2.076950	-1.381128	-1.760878
C	3.314166	-2.223619	-1.477463
H	3.024507	-3.172405	-1.022036
H	3.837064	-2.427713	-2.414343
H	4.004076	-1.700977	-0.808939
H	2.320978	-0.424507	-2.231344
H	1.376942	-1.906800	-2.414330
C	2.778544	0.776036	0.457987
C	2.833066	1.606524	1.560057
C	1.873914	1.535908	2.598810
C	0.829259	0.632129	2.570520
H	-1.227114	-1.536397	2.818893
H	-1.675424	-2.512294	1.412586
H	-2.275809	0.487628	1.826183
H	-3.445404	-0.832041	1.771490
H	0.205575	3.767339	0.175391
H	-0.874227	-0.062963	-2.780015
C	0.375872	1.671128	-2.490430
H	0.900485	1.539480	-3.432419
H	1.480709	3.432048	-1.929401
H	3.511200	0.833153	-0.339579
H	1.966775	2.211055	3.442419
H	3.633378	2.334273	1.636423
H	-0.438240	-2.242581	-0.742871
H	0.097332	0.580140	3.369386
O	0.897887	-3.549289	0.431366
H	1.508204	-3.347709	1.170922

Table S7. Coordinates of the structures the RAF radicals at each available site of **(1·H)**$^{+}$. Level of theory: M06-2X/6-31G(d).

C2

C	0.063805	2.844069	-0.547995
C	0.689078	2.587961	-1.769210
C	-0.774796	0.699283	-2.109384
C	-1.386717	0.943617	-0.878182
C	-0.984443	2.039953	-0.112263
H	-1.492740	2.277491	0.817776
Se	-2.820095	-0.206925	-0.350054
C	-2.404426	-0.446636	1.544139
C	-1.319099	-1.490559	1.813180
N	-0.066637	-1.168821	1.149863
C	0.769962	-0.167967	1.466660
C	1.752809	-0.067043	0.422240
N	1.469800	-0.992636	-0.501979
C	0.303109	-1.778546	-0.124766
C	2.257492	-1.304016	-1.689845
C	3.482226	-2.150544	-1.356136
H	3.179235	-3.094071	-0.898262
H	4.043996	-2.362080	-2.268870
H	4.141830	-1.622723	-0.661652
H	2.531421	-0.351566	-2.154813
H	1.592226	-1.824244	-2.384873
C	2.782549	0.891333	0.484538
C	2.792499	1.726171	1.577845
C	1.809077	1.634468	2.605434
C	0.794678	0.701928	2.568337
H	-1.125177	-1.576677	2.888344
H	-1.627484	-2.476215	1.455796
H	-2.140813	0.522743	1.972636
H	-3.340972	-0.769116	2.004540
H	0.378023	3.688587	0.057655
H	-1.114228	-0.131062	-2.724620
C	0.255125	1.526011	-2.557292
H	0.709503	1.340968	-3.526197
H	1.492188	3.231398	-2.114010
H	3.528463	0.963398	-0.298911
H	1.869636	2.319385	3.444253
H	3.568926	2.477946	1.667645
H	-0.516655	-1.630974	-0.841736
H	0.055778	0.631928	3.358901
O	0.625428	-3.115889	0.009125
H	0.026930	-3.659243	-0.523754

C6 (R)

C	0.040867	2.858015	0.627339
C	0.786824	3.049009	-0.537140
C	-0.540560	1.364864	-1.652528
C	-1.302602	1.194370	-0.494713
C	-1.016395	1.953446	0.641751
H	-1.632065	1.866415	1.532057
Se	-2.807913	0.018242	-0.586202
C	-2.676867	-0.862239	1.148722
C	-1.787764	-2.104993	1.107426
N	-0.460211	-1.779504	0.591951
C	0.518385	-1.063430	1.263402
C	1.511377	-0.801463	0.346304

N	1.107408	-1.333465	-0.857771
C	-0.087020	-1.899017	-0.685161
C	1.823242	-1.249387	-2.146020
C	3.166569	-1.959329	-2.105522
H	3.059810	-2.994574	-1.772433
H	3.593226	-1.963326	-3.111136
H	3.867158	-1.432804	-1.454143
H	1.944763	-0.187247	-2.366927
H	1.157216	-1.692026	-2.890470
C	2.779211	-0.075239	0.666110
C	2.654916	0.529220	2.043314
C	1.640132	0.255583	2.923877
C	0.547504	-0.576013	2.596720
H	-1.663492	-2.542865	2.102305
H	-2.214805	-2.866996	0.452357
H	-2.323047	-0.143896	1.890575
H	-3.694295	-1.151779	1.418209
H	0.255583	3.446187	1.513838
H	-0.773724	0.783018	-2.541009
C	0.492502	2.300785	-1.677844
H	1.058800	2.454749	-2.591976
H	1.575835	3.795524	-0.562778
H	-0.235163	-0.801438	3.310540
H	1.669469	0.701626	3.912529
H	-0.680267	-2.359078	-1.461643
O	3.138950	0.853071	-0.334055
H	3.616098	-0.791965	0.685200
H	3.473563	1.180819	2.331597
H	2.470480	1.560554	-0.328869

C6 (S)

C	-0.112685	2.772828	-0.807297
C	0.554659	2.391836	-1.971214
C	-0.814185	0.406480	-2.096783
C	-1.481014	0.787449	-0.930748
C	-1.145032	1.987175	-0.301790
H	-1.694903	2.323026	0.572696
Se	-2.907120	-0.327451	-0.311194
C	-2.548920	-0.316663	1.606272
C	-1.538954	-1.384352	2.026946
N	-0.284316	-1.231166	1.292809
C	0.637789	-0.211245	1.469951
C	1.527129	-0.300676	0.423489
N	1.127347	-1.354149	-0.363204
C	0.028466	-1.886199	0.171021
C	1.814018	-1.838748	-1.573431
C	2.832309	-2.920888	-1.247192
H	2.353839	-3.782165	-0.773097
H	3.307657	-3.258377	-2.170871
H	3.603768	-2.520605	-0.587231
H	2.288345	-0.965310	-2.026201
H	1.037490	-2.193372	-2.255831
C	2.687508	0.614458	0.208702
C	2.683757	1.662485	1.291483
C	1.781969	1.717003	2.323406
C	0.720350	0.796410	2.465752
H	-1.311792	-1.323114	3.095539
H	-1.920432	-2.385535	1.816776
H	-2.220108	0.680591	1.905394
H	-3.507016	-0.514612	2.090827
H	0.146603	3.701873	-0.309521
H	-1.095815	-0.516430	-2.598481
C	0.194489	1.212685	-2.621452
H	0.689119	0.924203	-3.544441
H	1.336994	3.023100	-2.381394
H	0.025718	0.848262	3.295051
H	1.887382	2.498351	3.068848
H	-0.541527	-2.702957	-0.247847
H	2.587832	1.102690	-0.771327
H	3.487562	2.389237	1.231436
O	3.921441	-0.086070	0.126923
H	4.178709	-0.349988	1.024948

C7 (R)

C	-0.396726	-2.782605	-0.818782
C	-1.053382	-2.260983	-1.934544
C	0.571682	-0.478763	-2.044587
C	1.220133	-0.993067	-0.918314
C	0.744279	-2.158663	-0.318553
H	1.277955	-2.604723	0.515637
Se	2.830418	-0.140756	-0.335764
C	2.525248	-0.114879	1.592080
C	1.801180	1.151141	2.053771
N	0.553347	1.329399	1.314114
C	-0.581784	0.560475	1.455582
C	-1.425142	0.876540	0.378027
N	-0.736838	1.827675	-0.380817
C	0.438164	2.057251	0.196105
C	-1.251693	2.465677	-1.596411
C	-2.321000	3.502837	-1.279752
H	-1.909817	4.311136	-0.670353
H	-2.700720	3.930415	-2.210226
H	-3.159605	3.053740	-0.741598
H	-1.636881	1.659625	-2.228331
H	-0.396812	2.911810	-2.110718
C	-2.643665	0.269966	0.173543
C	-3.093278	-0.788434	1.146483
C	-2.113997	-1.056950	2.269214
C	-0.927145	-0.431889	2.423889
H	1.558588	1.109365	3.119540
H	2.414150	2.037670	1.879192
H	1.973337	-1.013532	1.874904

H	3.510853	-0.161206	2.058770
H	-0.744397	-3.705009	-0.360596
H	0.965720	0.411555	-2.529251
C	-0.559057	-1.112913	-2.552488
H	-1.043702	-0.722624	-3.442588
H	-1.926471	-2.764156	-2.339595
H	-0.255824	-0.675030	3.241429
H	-2.419730	-1.832882	2.964103
H	1.202610	2.719281	-0.184118
H	-3.281206	0.486201	-0.676175
O	-3.415862	-1.991871	0.469402
H	-4.047280	-0.475719	1.596623
H	-2.606559	-2.283210	0.019459

C7 (S)

C	-0.235849	-2.384742	-1.560479
C	-0.763845	-1.598532	-2.584030
C	0.880452	0.105690	-2.110783
C	1.399997	-0.676919	-1.077042
C	0.856257	-1.936520	-0.822618
H	1.290866	-2.581197	-0.064238
Se	2.922780	-0.011451	-0.128663
C	2.443829	-0.506849	1.697220
C	1.600814	0.560863	2.396630
N	0.414322	0.880098	1.604143
C	-0.662172	0.044299	1.398365
C	-1.419350	0.603039	0.355912
N	-0.749371	1.770497	-0.020465
C	0.345763	1.885920	0.723280
C	-1.194577	2.694347	-1.068264
C	-2.370428	3.544907	-0.606476
H	-2.088163	4.170045	0.244019
H	-2.691911	4.196103	-1.422148
H	-3.218217	2.920395	-0.313200
H	-1.449337	2.076772	-1.934676
H	-0.333434	3.309787	-1.340075
C	-2.557742	0.014243	-0.145414
C	-3.000377	-1.304370	0.432990
C	-2.146382	-1.797227	1.581126
C	-1.045226	-1.169894	2.047381
H	1.267990	0.227343	3.383862
H	2.167070	1.485796	2.522788
H	1.931795	-1.470950	1.684618
H	3.386496	-0.632980	2.233040
H	-0.652101	-3.366846	-1.356412
H	1.324770	1.074910	-2.325826
C	-0.191796	-0.360059	-2.868878
H	-0.572778	0.241149	-3.689488
H	-1.599034	-1.962091	-3.174509
H	-0.478055	-1.559839	2.886794
H	-2.494609	-2.714461	2.046192
H	1.091755	2.660798	0.621029
H	-3.146559	0.443270	-0.948187
H	-2.954917	-2.059383	-0.367898
O	-4.370631	-1.284754	0.793445
H	-4.481966	-0.654587	1.522040

C8 (R)

C	0.133232	-0.812465	2.766149
C	0.473778	0.491413	3.125153
C	-1.159458	1.338040	1.561024
C	-1.489581	0.033484	1.191413
C	-0.859960	-1.043402	1.818890
H	-1.156142	-2.062784	1.587684
Se	-2.885467	-0.211632	-0.093864
C	-2.101649	-1.622806	-1.189104
C	-1.222142	-1.077255	-2.315650
N	-0.175125	-0.209851	-1.785067
C	0.917387	-0.638075	-1.025952
C	1.489748	0.526766	-0.486471
N	0.725109	1.587254	-0.919376
C	-0.272922	1.115964	-1.668444
C	0.975215	3.002438	-0.613953
C	2.120796	3.563159	-1.445542
H	1.888142	3.513825	-2.512018
H	2.287240	4.608637	-1.177020
H	3.047900	3.012325	-1.266586
H	1.177057	3.055393	0.459349
H	0.040956	3.537089	-0.801585
C	2.656601	0.487327	0.337225
C	3.166262	-0.729067	0.625491
C	2.584822	-2.042437	0.150319
C	1.407890	-1.899535	-0.778858
H	-0.738022	-1.885909	-2.871562
H	-1.812272	-0.486491	-3.019295
H	-1.542804	-2.305675	-0.546451
H	-2.940456	-2.173658	-1.618716
H	0.616721	-1.654406	3.252547
H	-1.673332	2.174801	1.093078
C	-0.186390	1.565029	2.533842
H	0.043932	2.582279	2.837635
H	1.233224	0.666582	3.880135
H	3.109133	1.400544	0.709682
H	4.058456	-0.821595	1.237214
H	-1.062702	1.715389	-2.098052
H	0.987434	-2.800960	-1.210941
O	3.586316	-2.879618	-0.398259
H	2.249540	-2.595045	1.043489
H	3.949690	-2.441215	-1.183228

C8 (S)

C	-0.009699	-2.599684	1.569626

C	0.728868	-1.895827	2.522004
C	-0.594486	0.073535	2.075171
C	-1.313067	-0.620630	1.101022
C	-1.034399	-1.969167	0.866407
H	-1.640192	-2.537486	0.165725
Se	-2.763719	0.263405	0.221847
C	-2.352864	-0.197607	-1.629923
C	-1.486474	0.858859	-2.319790
N	-0.263944	1.100478	-1.562220
C	0.792308	0.193843	-1.431276
C	1.601701	0.694215	-0.397244
N	1.004535	1.850160	0.055664
C	-0.115274	2.053890	-0.641274
C	1.524864	2.718345	1.120437
C	2.718351	3.536262	0.646225
H	2.434605	4.195204	-0.177806
H	3.087326	4.151990	1.469370
H	3.533309	2.890333	0.309389
H	1.781419	2.063095	1.957971
H	0.699161	3.358484	1.440317
C	2.766464	0.004214	0.058488
C	3.065282	-1.165468	-0.546277
C	2.233479	-1.810409	-1.632821
C	1.056883	-0.986186	-2.086402
H	-1.200812	0.543203	-3.327743
H	-2.018340	1.809326	-2.398950
H	-1.869295	-1.176700	-1.645744
H	-3.309045	-0.279983	-2.149593
H	0.178305	-3.659045	1.411419
H	-0.844131	1.110931	2.286078
C	0.423292	-0.563616	2.784568
H	0.964216	-0.022615	3.555707
H	1.515141	-2.396058	3.077610
H	3.370196	0.401151	0.868088
H	3.924981	-1.744615	-0.224271
H	-0.808769	2.865815	-0.476356
H	0.427267	-1.385887	-2.873352
H	2.882306	-2.016345	-2.495007
O	1.800429	-3.096372	-1.211634
H	1.239848	-2.957451	-0.430310

C9 (R)

C	-0.075220	-0.302628	-2.916254
C	0.590012	-1.519743	-2.773617
C	-1.420362	-0.799929	-0.971579
C	-1.095567	0.044963	-2.035465
H	-1.652507	0.964542	-2.194038
Se	-2.796267	-0.361110	0.283119
C	-2.334985	1.505446	0.621406
C	-1.249145	1.683076	1.684091
N	-0.079913	0.846003	1.398036
C	0.879240	1.091405	0.444484
C	1.691240	-0.017823	0.387372
N	1.188403	-0.923118	1.309768
C	0.119300	-0.380169	1.889238
C	1.749915	-2.248625	1.596019
C	3.017009	-2.153714	2.435046
H	2.807423	-1.695854	3.404742
H	3.417415	-3.155393	2.605745
H	3.783152	-1.560099	1.929278
H	1.930423	-2.724027	0.627635
H	0.971114	-2.821167	2.105462
C	2.799578	-0.113817	-0.493500
C	3.045532	1.022432	-1.298371
C	2.258124	2.143023	-1.270262
C	1.039142	2.312818	-0.400545
H	-0.898724	2.717969	1.723609
H	-1.619488	1.391366	2.668815
H	-2.055920	1.981547	-0.319782
H	-3.254102	1.981132	0.969322
H	0.178340	0.364277	-3.734381
C	0.224129	-2.391012	-1.750518
H	1.370407	-1.798208	-3.474358
H	3.424535	-0.996589	-0.540756
H	3.901807	1.002868	-1.964486
H	-0.509936	-0.861340	2.623714
C	-0.770483	-2.028045	-0.843067
H	0.705970	-3.360673	-1.661407
H	-1.048783	-2.700611	-0.034840
O	1.060399	3.501496	0.374631
H	0.155566	2.432845	-1.046195
H	2.493421	2.993073	-1.902762
H	1.925583	3.563984	0.810097

C9 (S)

C	-0.421139	2.238138	-2.024731
C	0.498013	1.520872	-2.791705
C	-1.384917	0.167345	-1.230544
C	-1.375628	1.564901	-1.265453
H	-2.133496	2.123785	-0.721519
Se	-2.657499	-0.784353	-0.166292
C	-2.385666	0.157267	1.522168
C	-1.254852	-0.418644	2.378192
N	-0.019106	-0.614125	1.612947
C	0.921717	0.339310	1.290833
C	1.802361	-0.241818	0.407274
N	1.369041	-1.544800	0.213773
C	0.268966	-1.732428	0.939654
C	2.024074	-2.542796	-0.640124
C	3.297552	-3.080429	-0.001422
H	3.075001	-3.585007	0.941752
H	3.768070	-3.799573	-0.675402

H	4.011721	-2.276559	0.195479
H	2.220312	-2.053027	-1.598546
H	1.296018	-3.338192	-0.817059
C	2.896665	0.456115	-0.167231
C	3.044719	1.803045	0.230992
C	2.204685	2.410459	1.126450
C	1.009738	1.752514	1.770366
H	-1.025323	0.254607	3.208295
H	-1.529431	-1.392609	2.788491
H	-2.203852	1.212251	1.321362
H	-3.324936	0.064842	2.070748
H	-0.421102	3.324166	-2.050991
C	0.438942	0.130624	-2.816882
H	1.230530	2.047416	-3.394433
H	3.566400	-0.009047	-0.879484
H	3.861634	2.377790	-0.192550
H	-0.325565	-2.633825	0.957375
C	-0.496925	-0.546073	-2.034130
H	1.115861	-0.430933	-3.454649
H	-0.541676	-1.632651	-2.048410
H	1.121059	1.773940	2.864358
O	-0.187498	2.477793	1.527874
H	2.356300	3.449175	1.401084
H	-0.241954	2.611147	0.565746

C13

C	0.242178	1.086643	2.432918
C	0.420645	2.395466	1.904850
C	-1.259833	1.937945	0.097690
C	-1.339525	0.550787	0.686073
C	-0.661028	0.193932	1.819712
H	-0.806509	-0.784300	2.269711
Se	-2.581181	-0.536916	-0.246641
C	-1.788659	-2.300713	0.028566
C	-0.716463	-2.624055	-1.015482
N	0.338981	-1.611834	-1.023965
C	1.343584	-1.473717	-0.069757
C	1.943020	-0.231879	-0.300869
N	1.269685	0.339368	-1.377607
C	0.320635	-0.505184	-1.768061
C	1.600727	1.628174	-2.002447
C	2.885706	1.546363	-2.815145
H	2.783815	0.827860	-3.631991
H	3.106072	2.526582	-3.243466
H	3.732948	1.246729	-2.193207
H	1.667420	2.356814	-1.188567
H	0.748646	1.906017	-2.627920
C	2.981072	0.245964	0.498820
C	3.382937	-0.575601	1.538951
C	2.776785	-1.826265	1.774426
C	1.747545	-2.301508	0.978095
H	-0.245120	-3.591967	-0.821876
H	-1.149411	-2.653381	-2.017545
H	-1.398862	-2.383091	1.044412
H	-2.609823	-3.013159	-0.072926
H	0.772572	0.783228	3.327860
C	-0.273535	2.812771	0.813006
H	-0.183266	3.829422	0.440033
H	1.099865	3.077949	2.407022
H	1.283784	-3.264686	1.163042
H	3.439803	1.213381	0.325275
H	3.129263	-2.433979	2.600525
H	4.186209	-0.250027	2.190625
H	-0.391753	-0.314222	-2.559230
O	-2.555945	2.525554	0.026101
H	-0.987815	1.859151	-0.967708
H	-2.865517	2.664908	0.935870

C14

C	-0.264633	2.515551	1.583125
C	0.700401	2.919384	0.709829
C	-0.338445	1.468555	-1.041805
C	-1.269507	1.076287	-0.132088
C	-1.276180	1.608891	1.189704
H	-2.085693	1.366284	1.869062
Se	-2.663908	-0.121654	-0.672700
C	-2.539093	-1.384150	0.810331
C	-1.528415	-2.502662	0.554373
N	-0.217430	-1.965422	0.195950
C	0.675659	-1.330061	1.054165
C	1.691151	-0.802794	0.249544
N	1.362429	-1.124246	-1.065236
C	0.215563	-1.794557	-1.052057
C	2.163286	-0.807335	-2.256186
C	3.389924	-1.703270	-2.362808
H	3.098185	-2.751780	-2.459792
H	3.968340	-1.422403	-3.245593
H	4.033707	-1.601785	-1.485396
H	2.434868	0.249903	-2.186466
H	1.504665	-0.920607	-3.120542
C	2.759103	-0.083680	0.789289
C	2.751083	0.089462	2.163448
C	1.725635	-0.438797	2.973223
C	0.670343	-1.158978	2.438975
H	-1.398544	-3.135265	1.437637
H	-1.854092	-3.137769	-0.272011
H	-2.293367	-0.830801	1.719075
H	-3.532646	-1.818620	0.934899
H	-0.283862	2.915565	2.592400
H	-0.365106	1.098205	-2.064598
C	0.746567	2.457497	-0.716355
H	-0.115977	-1.566665	3.065445

H	3.551367	0.321308	0.168132
H	1.766964	-0.277925	4.044769
H	3.557409	0.643704	2.630796
H	-0.308287	-2.141323	-1.932162
O	0.764299	3.541975	-1.634625
H	1.724190	1.975131	-0.891630
H	1.458946	3.638008	1.004839
H	-0.052226	4.048893	-1.503305

C15

C	0.876344	2.368502	1.006994
C	1.834848	2.471148	-0.145245
C	-0.000474	1.403965	-1.496060
C	-0.836414	1.316872	-0.346352
C	-0.375324	1.856273	0.889811
H	-1.045429	1.880813	1.745045
Se	-2.587360	0.645211	-0.571709
C	-2.788251	-0.396719	1.069067
C	-2.282761	-1.833425	0.910844
N	-0.902883	-1.847321	0.432471
C	0.209804	-1.442371	1.165626
C	1.257017	-1.299479	0.250044
N	0.736378	-1.616181	-1.001564
C	-0.549348	-1.909068	-0.852596
C	1.492992	-1.630212	-2.260092
C	2.404945	-2.845678	-2.355887
H	1.824246	-3.771115	-2.342429
H	2.966751	-2.804433	-3.291589
H	3.119586	-2.872727	-1.529312
H	2.048765	-0.688868	-2.294338
H	0.760681	-1.609913	-3.070947
C	2.525594	-0.873602	0.643596
C	2.689607	-0.594586	1.990992
C	1.635559	-0.745655	2.914648
C	0.377838	-1.174352	2.524278
H	-2.316077	-2.375834	1.860344
H	-2.886764	-2.382394	0.185416
H	-2.274182	0.117767	1.882946
H	-3.856167	-0.408803	1.294332
H	1.211842	2.797909	1.946555
H	-0.388579	1.051887	-2.450359
C	1.253482	1.926413	-1.419121
H	-0.430725	-1.290247	3.238316
H	3.338885	-0.760764	-0.065872
H	1.817291	-0.521663	3.959969
H	3.656666	-0.256496	2.347103
H	-1.226327	-2.148559	-1.660834
H	1.869376	2.025043	-2.309266
O	2.289817	3.807395	-0.317436
H	2.757737	1.924394	0.101624
H	1.516922	4.354020	-0.528724

C16

C	0.305034	-2.610735	-0.119668
C	0.795150	-2.587147	1.298987
C	-0.796520	-0.875064	1.934828
C	-1.331060	-0.848616	0.616916
C	-0.856435	-1.683284	-0.347308
Se	-2.785160	0.365720	0.310944
C	-2.349107	0.970999	-1.491039
C	-1.333082	2.112783	-1.501469
N	-0.096738	1.736144	-0.817046
C	0.916064	0.929786	-1.329043
C	1.783865	0.660831	-0.264768
N	1.254806	1.303045	0.850346
C	0.134524	1.914372	0.483853
C	1.856009	1.317354	2.190077
C	3.035516	2.277233	2.266078
H	2.717308	3.303117	2.065379
H	3.470071	2.240829	3.267448
H	3.812748	2.008134	1.546105
H	2.145401	0.285680	2.407766
H	1.063149	1.590641	2.890711
C	2.926076	-0.125609	-0.424447
C	3.149821	-0.633389	-1.693930
C	2.268339	-0.373330	-2.763753
C	1.136647	0.410204	-2.605407
H	-1.068042	2.405172	-2.521808
H	-1.733648	2.991154	-0.991317
H	-2.007814	0.123895	-2.088028
H	-3.289467	1.325072	-1.918507
H	-1.219359	-0.231608	2.700384
C	0.247856	-1.780789	2.251286
H	0.602377	-1.833091	3.277324
H	1.584463	-3.291671	1.543861
H	0.462880	0.607971	-3.432260
H	3.599422	-0.332981	0.400331
H	2.484050	-0.799098	-3.737375
H	4.022982	-1.251553	-1.871036
H	-0.523409	2.457104	1.148765
H	-1.310256	-1.742248	-1.332861
H	1.127073	-2.310014	-0.790870
O	0.002912	-3.930083	-0.549447
H	-0.706435	-4.268235	0.019238

C17

C	-0.688286	2.975858	-0.064786
C	-0.008629	3.160396	-1.225606
C	-0.507247	0.868713	-1.931175
C	-1.193056	0.629953	-0.767310
C	-1.275198	1.653242	0.341911
Se	-2.263415	-0.924191	-0.646718

C	-2.056596	-1.371647	1.240409
C	-0.874989	-2.318536	1.484890
N	0.355528	-1.768911	0.918296
C	1.046339	-0.671791	1.427913
C	1.880936	-0.214934	0.403522
N	1.673939	-1.061343	-0.683111
C	0.746299	-1.949152	-0.345855
C	2.372159	-0.974493	-1.971677
C	3.806351	-1.475809	-1.871062
H	3.830874	-2.529778	-1.584123
H	4.297107	-1.370731	-2.841106
H	4.376690	-0.902923	-1.135395
H	2.320106	0.074291	-2.279218
H	1.790155	-1.558922	-2.688556
C	2.681493	0.917316	0.556347
C	2.612595	1.557343	1.783343
C	1.787430	1.084630	2.823392
C	0.988300	-0.037354	2.669303
H	-0.708283	-2.485416	2.552792
H	-1.047576	-3.288980	1.014771
H	-1.983353	-0.441193	1.807737
H	-2.982250	-1.870675	1.532911
H	-0.484773	0.100446	-2.702294
C	0.128391	2.104497	-2.168134
H	0.651002	2.271429	-3.103474
H	0.413568	4.133436	-1.456444
H	0.344531	-0.391435	3.467484
H	3.316142	1.283885	-0.243157
H	1.774980	1.616331	3.768329
H	3.212581	2.444772	1.950923
H	0.341769	-2.701596	-1.007775
H	-0.693598	1.296302	1.209106
O	-2.586214	1.769018	0.872001
H	-0.822384	3.782496	0.649687
H	-3.185487	1.990993	0.141365

Table S8. Coordinates of the structures for the Se oxidation of **(1·H)**$^{+}$. Level of theory: M06-2X/6-31G(d).

TS

C	-0.319652	2.686302	-0.604854
C	0.547943	2.612821	-1.694458
C	-0.096449	0.316965	-2.057867
C	-0.945438	0.393487	-0.954150
C	-1.089468	1.584963	-0.242741
H	-1.834319	1.661131	0.545010
Se	-2.002606	-1.129461	-0.519624
C	-1.763463	-1.218990	1.403952
C	-0.521107	-1.992352	1.827094
N	0.675037	-1.430839	1.200977
C	1.249492	-0.198919	1.502786
C	2.190839	0.052028	0.499492
N	2.143486	-1.032384	-0.371409
C	1.218381	-1.876466	0.068022
C	2.994421	-1.208550	-1.554796
C	4.416121	-1.597312	-1.172346
H	4.430920	-2.557341	-0.650734
H	5.023781	-1.685526	-2.075721
H	4.872489	-0.843826	-0.525073
H	2.958739	-0.262877	-2.103094
H	2.520820	-1.971207	-2.178184
C	2.950697	1.222113	0.477279
C	2.719574	2.122601	1.502689
C	1.769236	1.870452	2.513484
C	1.016146	0.708756	2.537152
H	-0.382730	-1.953707	2.911584
H	-0.582984	-3.042343	1.532007
H	-1.770208	-0.190058	1.767594
H	-2.667190	-1.710700	1.769859
H	-0.424048	3.615857	-0.054485
H	-0.032364	-0.606338	-2.629938
C	0.644863	1.435624	-2.433421
H	1.280423	1.393174	-3.313307
H	1.125681	3.484428	-1.985448
H	3.676570	1.419577	-0.304292
H	1.625392	2.608680	3.294701
H	3.283435	3.048491	1.530783
H	0.932847	-2.795469	-0.426397
H	0.284315	0.520927	3.315709
O	-3.646343	0.067759	0.051424
O	-4.870475	1.479472	0.198201
H	-5.645906	0.919659	0.363835
H	-4.074988	0.732721	-0.625078

5

C	0.228287	-2.842716	-0.468564
C	0.933913	-2.783862	0.735196
C	-0.670067	-1.211697	1.615878
C	-1.348666	-1.258801	0.403045
C	-0.932844	-2.094472	-0.630240
H	-1.547484	-2.177678	-1.524383
Se	-3.011076	-0.307223	0.170773
C	-2.434833	0.900358	-1.272278
C	-1.535725	2.063527	-0.896170
N	-0.236877	1.610856	-0.384661
C	0.708143	0.876640	-1.095720
C	1.731134	0.572333	-0.192011

N	1.371085	1.142200	1.023824
C	0.194792	1.737624	0.868330
C	2.167832	1.085115	2.256013
C	3.351848	2.041409	2.204105
H	3.013547	3.076546	2.115262
H	3.935760	1.947161	3.122368
H	4.005869	1.815874	1.357798
H	2.484233	0.044260	2.367808
H	1.494831	1.317658	3.085290
C	2.848106	-0.177078	-0.565744
C	2.889704	-0.601730	-1.881683
C	1.865442	-0.283528	-2.797908
C	0.758628	0.460330	-2.428529
H	-1.342739	2.706988	-1.760412
H	-1.989909	2.681550	-0.116488
H	-2.007336	0.239067	-2.029817
H	-3.387833	1.277638	-1.652066
H	0.568913	-3.495160	-1.266228
H	-1.054668	-0.608688	2.436835
C	0.479198	-1.985430	1.782635
H	0.993314	-1.994901	2.739646
H	1.822224	-3.393819	0.868405
H	3.638000	-0.418922	0.137404
H	1.949501	-0.630363	-3.821939
H	3.735194	-1.189749	-2.221553
H	-0.342717	2.253697	1.653089
H	-0.019797	0.710087	-3.141030
O	-3.895874	-1.340664	-0.768584

Table S9. Coordinates of the structures of the five ROSs and their protonated forms. Level of theory: M06-2X/6-31G(d).

$^{\bullet}$OH

O	-0.006151	0.000000	-1.510008
H	-0.006151	0.000000	-2.488867

HOH

O	0.019792	-0.005769	-1.526814
H	0.002994	-0.002034	-2.492171
H	-0.889189	0.196357	-1.271714

$^{\bullet}$OOH

O	0.055503	0.709875	0.000000
O	0.055503	-0.601901	0.000000
H	-0.888054	-0.863793	0.000000

HOOH

O	0.000000	0.713705	-0.055572
O	0.000000	-0.713705	-0.055572
H	-0.812252	-0.891397	0.444578
H	0.812252	0.891397	0.444578

$^{\bullet}OCH_3$

C	-0.009738	-0.577810	0.000000
H	1.056999	-0.867510	0.000000
H	-0.460335	-1.006509	0.905992
H	-0.460335	-1.006509	-0.905992
O	-0.009738	0.793424	0.000000

$HOCH_3$

C	-0.046785	0.658091	0.000000
H	-1.091646	0.973590	0.000000
H	0.437523	1.077763	0.891937
H	0.437523	1.077763	-0.891937
O	-0.046785	-0.753213	0.000000
H	0.871595	-1.051960	0.000000

$^{\bullet}OOCH_3$

O	1.169320	-0.278307	0.000017
O	0.157975	0.542855	-0.000016
C	-1.081506	-0.181434	-0.000021
H	-1.130743	-0.801366	-0.896590
H	-1.131372	-0.800256	0.897305
H	-1.867213	0.573839	-0.000590

$HOOCH_3$

O	-1.147069	0.270796	0.102545
O	-0.025279	-0.605400	0.019773
H	-1.569086	0.104132	-0.756207
C	1.115476	0.222340	-0.021549
H	1.963984	-0.466065	-0.031970
H	1.125724	0.842163	-0.925757
H	1.165302	0.862565	0.864682

$^{\bullet}OOCH{=}CH_2$

C	-1.389731	-0.500063	0.000186
C	-0.628608	0.583193	-0.000384
H	-0.954065	-1.490586	0.001155
H	-2.466422	-0.390841	-0.000307
H	-0.972696	1.610046	-0.001325
O	0.762674	0.580046	0.000699
O	1.300227	-0.608472	-0.000491

$HOOCH{=}CH_2$

C	1.406517	-0.554260	0.001289
C	0.696641	0.565615	0.013829
H	0.940238	-1.527673	-0.060358
H	2.485961	-0.488479	0.033521

H	1.124964	1.562825	0.054755
O	-0.663060	0.702224	-0.016204
O	-1.287489	-0.566962	-0.101344
H	-1.565717	-0.696899	0.821765
